# Maternal Pre-Pregnancy Body Mass Index and Its Impact on Short- and Long-Chain Fatty Acid and Microbiome Profiles of Human Breast Milk in Caucasian Women of Northeast Tennessee

**DOI:** 10.3390/nu18121917

**Published:** 2026-06-12

**Authors:** Kristy L. Thomas, Amy E. Wahlquist, William Andrew Clark

**Affiliations:** 1 Department of Biomedical Science, Quillen College of Medicine, East Tennessee State University, Johnson City, TN 37614, USA; thomkris@musc.edu; 2 Department of Rehabilitative Science, College of Health Science, East Tennessee State University, Johnson City, TN 37614, USA; 3 Center for Rural Health and Research, Department of Biostatistics and Epidemiology, College of Public Health, East Tennessee State University, Johnson City, TN 37614, USA; wahlquist@etsu.edu

**Keywords:** body mass index, breast milk, short-chain fatty acids, long-chain fatty acids, microbiome, macronutrients

## Abstract

**Background**: Increasing evidence suggests that breast milk and its bioactive components, including short-chain fatty acids and the milk microbiome, are influenced by maternal nutrition and body mass index (BMI). Bioactive components transferred to the infant through breast milk play a pivotal role in infant growth and development and have indications in the child’s future short- and long-term health outcomes. This study aimed to assess the impact of maternal pre-pregnancy BMI (PP-BMI) on human breast milk macronutrient composition, short- and long-chain fatty acid profiles, and breast milk microbiome profiles. **Approach**: This was an exploratory cohort study of forty-four lactating Caucasian women, two to fourteen weeks postpartum, divided into groups based on pre-pregnancy body mass index (BMI). Study participants signed informed consent, completed health and nutritional surveys, and provided a breast milk sample. Breast milk samples were subjected to proximate analysis, microbiome identification and short- and long-chain fatty acid extraction and analysis. **Results**: Maternal age, maternal physical activity, infant birth weight, and time of lactation at sample collection were not significantly different between the maternal PP-BMI groups. PP-BMI was significantly different between the two maternal groups. No significant differences were found between the maternal BMI groups concerning nutritional intake. No differences in breast milk microbiomes were observed in alpha diversity and beta diversity between the maternal PP-BMI groups. For long-chain fatty analysis in breast milk samples, myristic acid was significantly higher in the PP-BMI overweight/obese group while stearic acid was significantly higher in the PP-BMI normal-weight group. Butyric, valeric, and isocaproic acid concentrations in HBM were significantly higher in the PP-BMI normal-weight group and lower or undetectable in the PP-BMI overweight/obese group. **Conclusions**: Data from this exploratory cohort study indicate that maternal diet and pre-pregnancy BMI may be associated with differences in selected HBM fatty acids. There were no significant differences in microbiomes for alpha and beta diversity in breast milk between maternal PP-BMI groups; however, lower relative abundance was observed in the breast milk of the PP-BMI overweight/obese group. These findings should be interpreted in the context of the study’s limitations, including convenience recruitment from a Facebook group, the modest sample size, and restriction to Caucasian women from a single geographic region.

## 1. Introduction

Breastfeeding is the gold standard for providing infants with nutrition, protection, and developmental support. The United Nations International Children’s Emergency Fund (UNICEF) and the World Health Organization (WHO) recommend that infants up to the age of six months be exclusively breastfed and children up to two years of age have breast milk as a fundamental component of their nutrition [1]. Human breast milk (HBM) is an ever-changing biological fluid that provides infants with nutrition and has protective effects against pathogens [2,3,4]. Other benefits HBM affords infants are improved survival rates; reduced risk of developing childhood infections, including pneumonia and diarrhea; gastrointestinal and improved immune function regulation; and increased neurodevelopment [1,3,5]. In their review on breastfeeding, Victoria et al. reveal the protective effects of HBM in infants at reducing the risk of overweight, obesity, and diabetes in adulthood, preventing misalignment of teeth, protecting against childhood infections, and increasing intelligence [6]. Besides providing the infant with the benefits of protection against pathogens and aiding in infant growth and development, HBM has been indicated to prevent future obesity and related metabolic diseases (type 2 diabetes) in the infant [3,7,8,9,10,11,12,13]. Additionally, the benefits of breastfeeding, both short- and long-term, not only extend to the infant but also to the mother [14,15,16,17]. Breastfeeding reduces the risk of the mother developing breast, ovarian, and uterine cancers and promotes weight loss postpartum [6].

However, maternal HBM has variations in composition depending on maternal and infant factors. Changes can occur in the composition of breast milk due to the length of gestation, delivery mode, the time and length of lactation, maternal health and disease state, maternal diet, and maternal genetics [4].

The composition and volume of HBM are influenced by the maternal diet [18]. Despite differences in maternal diet due to different culture populations, the macronutrient composition of HBM is relatively constant [19]. Researchers have found an increase in fat concentration of HBM at different stages of lactation postpartum when lactating women eat increased amounts of proteins and carbohydrates as part of an isocaloric high-fat diet [20,21,22]. In a cohort study of pregnant women by Barrera et al., researchers found that the quality of fatty acids consumed during pregnancy and lactation is reflected in the fatty acid abundance and composition in HBM [23]. Conversely, in a systematic review of HBM and omega-3 supplementation, Amaral et al. discovered that, despite differences in the study methods used, study sample size, time the study subjects took the omega-3 supplement or the omega-3 source that was used by the study subjects, there was a positive correlation between omega-3 consumption and the concentration of omega-3 in HBM [24]. Pregnant women who consumed two servings of salmon a week from the twentieth week of gestation until delivery had higher concentrations of docosahexaenoic acid (DHA) (90%), eicosapentaenoic acid (EPA) (80%), and docosapentaenoic acid (DPA) (30%) in HBM on the fifth day after delivery than women who did not consume two servings of fish per week [25]. Further, women who were pregnant and supplemented with fish oil at twenty weeks gestation until delivery not only increased DHA, EPA, and DPA concentrations in HBM at early-lactation stages but had increased levels of DHA up to six months postpartum in HBM, and the consumption of fish oil supplements was positively associated with the DHA status of the infant at one year of age [26]. This is because the deposition of *n*-3 and other long-chain fatty acids from the maternal diet is stored in maternal body fat, and maternal body fat accounts for up to seventy percent of the fat source in breast milk [27,28,29,30].

The pervasiveness of obesity is steadily increasing in the US, and currently, there is no universal approach to preventing obesity [31]. Approximately 32% of women of childbearing age are obese, and 24% are overweight in the US [32]. Rates of obesity have increased in women of childbearing age from 2013 to 2016 in all educational, age, racial, and Hispanic ethnic groups. Additionally, race, ethnicity, and income groups have disproportional rates of obesity when compared to Caucasians [31]. Maternal adiposity and other maternal factors, including maternal health and nutrition status, have been shown to influence HBM composition and the HBM microbiome [32,33,34,35].

It is maternal nutrition that affects the growth and development of the infant in utero and later. However, maternal body composition, specifically maternal adiposity, has been shown to affect maternal HBM composition [36]. Increased risk of obesity and the transfer of obesity from mother to offspring has been shown in a murine study by Tsuduki et al. through milk-dependent mechanisms [37]. Reduced duration, late-onset lactogenesis, and early breastfeeding termination have been associated with maternal obesity [38,39,40]. In addition to breastfeeding complications being associated with maternal obesity, several studies have found that obese women have an increased risk of adverse birth outcomes, including preterm pregnancies [41,42,43].

Not only does HBM contain micronutrients for infant nutrition, but it also contains bioactive components that contribute toward infant development and potentially promote future health or disease outcomes. Nevertheless, the influence of bioactive components in HBM and their role in the infant needs to be better understood [7]. Two examples of bioactive components found in HBM are adiponectin and leptin, known as adipose tissue-derived hormones (adipokines) [8,44,45,46].

Absent in formula, adiponectin, an anti-diabetic hormone, and leptin, an appetite-controlling hormone, can regulate body composition and metabolism in the breastfed infant [5,9,10,11,12,13,32]. Studies in murine models found that maternal diet influences the concentration of adiponectin and leptin in milk and the growth and development of the offspring [47,48,49]. Other bioactive components in HBM are short-chain fatty acids (SCFAs), metabolic byproducts of gut bacteria fermenting non-digestible fibers, and other substrates. Short-chain fatty acids are taken up in the lumen of the intestines and serve as a carbon source for colonocytes, positively influencing the intestinal mucosa, and regulating hormone production in the gut, inflammation, and energy homeostasis [2,50,51].

Maternal diet influences the amount and types of SCFAs produced and passed on to the infant through HBM, which could be essential to infant growth [52,53]. Additionally, maternal microbiota, another bioactive component, is transmitted through HBM after birth and through direct contact during vaginal birth [54,55,56]. A study by Cabrera-Rubio et al. found that maternal BMI and weight gained during pregnancy affect the HBM microbiome, particularly microbial composition, and diversity, with obese mothers having less microbial diversity than normal-weight mothers in their HBM [34]. Consequently, the maternal HBM microbiome, now known to be a key element in maternally transferred phenotypes and infant diet, plays a pivotal role in infant growth and development, gut microbiome colonization, maturation of the immune system, and metabolic development and thus has an impact on infant health and disease development later in life [57,58,59,60].

Research problem: The relationship between maternal adiposity and HBM SCFAs and the microbiome has been limitedly explored. Researching HBM components, specifically HBM SCFAs and the microbiome, is fundamental in understanding how to provide the optimal support for the best short- and long-term developmental outcomes in infants. Elucidating the dynamic connections between HBM components and infants’ growth and developmental outcomes is vital to creating strategies to modify and optimize HBM and its components to provide infants with the best opportunities.

## 2. Objectives or Purpose

This study aimed to determine if maternal PP-BMI or diet was associated with differences in breast milk fatty acid and/or microbial composition.

Research questions: Is there a difference in the gross macronutrient composition of HBM from women in differing BMI groups? Do the SCFA and LCFA profiles differ in the HBM of normal-weight women compared to women who are overweight/obese? Do the microbiome profiles of HBM from women of a normal-weight BMI differ significantly from the microbiome profiles in HBM of overweight/obese women?

**Hypothesis** **1.** *Like previous studies, we do not anticipate any significant differences in the gross macronutrient composition of HBM from mothers of differing PP-BMIs.*

**Hypothesis** **2.** *We hypothesize that there will be significant differences in the SCFA and LCFA profiles, with greater amounts of acetic, propionic, and butyric SCFA levels and greater saturated, omega-6 fatty acid and lower omega-3 fatty acid LCFA levels in the HBM of overweight/obese women compared to the HBM of women who are of normal body weight.*

**Hypothesis** **3.** *We hypothesize that maternal HBM reflects the gut microbiome, with elevated maternal PP-BMI contributing to reduced microbial abundance and diversity in HBM as demonstrated in previous studies.*

## 3. Materials and Methods

### 3.1. Study Design and Subjects

Forty-four individuals (*n* = 44) were recruited from a Facebook breastfeeding support group page, BABE Breastfeeding Coalition of Tri-Cities, in 2015. Participants were excluded from the study if they had any breastfeeding disorder. All participants completed a health and demographic survey (Figure S1), the Block Fruit–Vegetable–Fiber Questionnaire (Figure S2), and the Block Dietary Fat Questionnaire (Figure S3). Equations (developed by Block et al.) were used for dietary intake calculations [61]. Participants also provided approximately four ounces of expressed breast milk.

To ensure standardization of mature milk samples, participants were of Caucasian descent between 2 and 14 weeks of lactation. The 44 participants were separated into two groups based on the WHO standardized BMI metrics [62]: the normal-weight group (*n* = 24) with normal PP-BMI (body mass index) between 18.5 and 24.9 kg/m^2^ and the overweight/obese group (*n* = 20) with overweight/obese PP-BMI greater than 25.0 kg/m^2^. The overweight/obese PP-BMI group comprised 5 overweight (BMI 25.0–29.9 kg/m^2^), 6 class-1 obese (BMI 30.0–34.9 kg/m^2^), 6 class-2 obese (BMI 35.0–39.9 kg/m^2^) and 3 severely obese (BMI > 40.0 kg/m^2^) women. Because of the limited number of participants in this group (overweight/obese PP-BMI) and that two of the overweight women had BMI > 28.5 kg/m^2^, we elected to merge overweight and obese into one group for statistical analysis. Participants were compensated with a $20 gift card.

### 3.2. Breast Milk Collection and Proximate Analysis

HBM samples were self-collected under real-world, non-observed conditions without standardized procedures for timing, breast side, foremilk/hindmilk status, cleaning, or pump sterilization. The results should be interpreted as reflecting expressed HBM collected in naturalistic settings and not strictly standardized HBM composition. Participants were instructed to immediately freeze their HBM samples in their home freezer and notify the research laboratory that the sample was ready. Research Assistants from the research laboratory met the participants and transported the HBM sample to the laboratory in a cooler with ice packs, and it was immediately transferred to a −80 C freezer. Breast milk samples remained frozen at −80 degrees Celsius until analysis.

Whole HBM samples were freeze-dried for at least 24 h at 0.077 mBar at −50 °C in the LABCONCO FreeZone 2.5 Freeze Dryer (Kansas City, MO, USA).

The energy content of each lyophilized HBM sample was quantified using bomb calorimetry (Parr 6200 Calorimeter, PARR Instrument Co., Moline, IL, USA) using a method previously described [63]. Samples were run in duplicate. Macronutrient composition of each lyophilized human breast milk (HBM) sample was determined by averaging two qualifying measurements. Protein concentration was quantified using the Kjeldahl method, fat concentration was assessed using the Soxhlet extraction method, inorganic content (ash) was determined using an ashing oven (700 °C for 12 h), and carbohydrate concentration was calculated by difference, subtracting the measured amounts of protein, fat, water, and ash from the total sample weight.

### 3.3. Breast Milk SCFA, LCFA, and Microbiome Analysis

Short-chain fatty acids (SCFAs) were extracted from lyophilized HBM (300 mg) using a separatory funnel to which 5 mL of hexane and 5 mL of volatile fatty acid (VFA) solution (oxalic acid (0.1 M/L) and sodium azide (40 mM/L)) were added. The funnel was gently rocked back and forth 50 times and allowed to sit on a ring stand for 10 min to allow separation between the fat- and water-soluble phases. The bottom phase was decanted into 50 mL Falcon tubes, frozen at −80 C, and freeze dried. Freeze dried samples were resuspended in 500 ul of VFA solution, vortexed until fully homogenized and centrifuged at 4000× *g* for 15 min. Further processing of the samples was performed using a modified procedure developed by Schwiertz et al. [64]. Samples were analyzed for short-chain volatile fatty acids using a Shimadzu GC2010 gas chromatograph (Shimadzu, Kyoto, Japan) equipped with a Phenomenex Zebron ZB-Wax Plus capillary column (part # 7HG-G013-11; Phenomenex, Torrance, CA, USA). Samples were run in duplicate, and the values for each were averaged.

Long-chain fatty acid (LCFA) extractions were performed on 30 mg lyophilized HBM using a modified AOAC 969.33 method [65]. Fatty acid analysis was performed using flame ionizing detector (FID) gas chromatography (Shimadzu GC-2010; Shimadzu Corporation, Kyoto, Japan) equipped with a Zebron ZB-WAX capillary column (30 m × 0.25 mm i.d., 0.25 μm film thickness; Phenomenex, Torrance, CA, USA). Fatty acids were identified by comparison with authentic standards and quantified as a percentage of total fat based on peak area normalization. Internal standard (C17) and hexane blanks were included in each run.

Genomic DNA extraction and quantification, as well as 16S rRNA V3–V4 amplicon generation, library preparation, and Illumina MiSeq sequencing of HBM microbiome samples were accomplished at the University of Tennessee (UT) Genomics Core facility as previously described [66]. We used 30 ng of DNA in the initial PCR and ran the cycle number to 35 to amplify the samples. The UT Genomics Core includes a reagent-only negative PCR control that was carried out through the entire library preparation process.

### 3.4. 16S rRNA Gene Sequencing and Microbiome Analysis

Operational Taxonomic Unit (OTU) clustering, taxonomic assignments, alpha and beta diversity analyses, PERMANOVA testing, and differential abundance modeling with TMM normalization were all performed using the CLC Genomics Workbench v. 23 and CLC Microbial Genomics Module v. 2.5 (Qiagen, Hilden, Germany) [67], using a previously described method [66].

### 3.5. Statistical Analysis

All statistical analyses were conducted using SAS 9.4 (SAS Institute, Cary, NC, USA), SPSS v29 (IBM Corp., Armonk, NY, USA), and the CLC Genomics Workbench v23 with the CLC Microbial Genomics Module v2.5 (Qiagen, Hilden, Germany). Descriptive statistics (means and standard deviations for normally distributed variables; medians and interquartile ranges for skewed variables; frequencies and percentages for categorical variables) were calculated separately for the normal-weight and overweight/obese PP-BMI groups.

Assessment of distributional assumptions: normality of continuous variables was evaluated using the Shapiro–Wilk test and inspection of Q–Q plots. Equality of variances was assessed using Levene’s test. These diagnostics informed the choice of parametric versus non-parametric tests.

Group comparisons for maternal, dietary, and milk composition variables: continuous variables (e.g., maternal anthropometrics, dietary intake, proximate composition, and long-chain fatty acids) were compared between PP-BMI groups using two-sample *t*-tests when normality and variance assumptions were met, or Wilcoxon rank-sum tests when distributions were skewed or sample sizes were small. Categorical variables (e.g., education, employment, income, prenatal vitamin use) were compared using Fisher’s exact tests due to small cell counts.

Short-chain fatty acids (SCFAs): SCFA concentrations were run in duplicate and averaged to create a single value per participant. Because several SCFAs exhibited zero-inflation and non-normal distributions, Wilcoxon rank-sum tests were used for all SCFA comparisons.

Long-chain fatty acids (LCFAs): LCFA values were expressed as a percent of total fat. Parametric or non-parametric tests were applied based on distributional characteristics. Multiple LCFAs were evaluated; therefore, Benjamini–Hochberg false discovery rate (FDR) correction was applied to control for multiple comparisons.

Microbiome analyses: 16S rRNA sequencing data were processed in CLC Genomics Workbench v. 23. Alpha diversity (Shannon, Chao1) and beta diversity (Bray–Curtis dissimilarity) were compared between PP-BMI groups using non-parametric tests and PERMANOVA, respectively. Differential abundance testing was performed using the ANOVA-like generalized linear model (GLM) implemented in CLC with trimmed mean of M-values (TMM) normalization and Benjamini–Hochberg FDR correction. Taxa with FDR-adjusted *p* < 0.05 were considered significantly different.

Missing data: missing values were minimal and handled using listwise deletion, consistent with the exploratory design and the small number of missing observations.

Statistical significance: two-sided *p*-values < 0.05 were considered statistically significant unless otherwise noted. For analyses involving multiple comparisons (LCFAs and microbiome), significance was based on FDR-adjusted *p*-values.

This experiment was designed as an exploratory research project to determine if there were any trends evident with respect to maternal PP-BMI and HBM nutrient and microbiome components. Because of the small cohort recruited for this project, corrections for probiotic use, maternal diabetes status, smoking, medications, exclusive breastfeeding status, infant sex, mastitis history and incidence of infections were not made.

## 4. Results

### 4.1. Subjects

Forty-four participants provided at least four ounces of expressed HBM and filled out a demographic and health survey (Figure S1) and two Block food frequency questionnaires (Figures S2 and S3). The study participants were separated into two groups: one group of women with a normal PP-BMI (*n* = 24) and one group with an overweight or obese PP-BMI (*n* = 20).

### 4.2. Subject Demographics

All participants in this study were Caucasian and non-Hispanic. The normal-weight and overweight/obese groups were of similar age, 28.79 ± 5.04 and 28.35 ± 4.21 years, respectively.

We obtained information about the participants’ marital status, education, employment, income status, infant birth weight (in ounces), and number of pregnancies (Table 1).

Most participants in both BMI groups were married, with the normal PP-BMI group having higher education levels and more full-time homemakers, while the overweight/obese PP-BMI group had more part-time workers and some unemployment.

Household income varied widely across both BMI groups, with the normal-weight PP-BMI group skewing toward mid-to-high income brackets and the overweight/obese PP-BMI group showing more participants in lower income ranges, though both had individuals earning over $100,000 annually.

Infants born to the normal-weight PP-BMI group were slightly smaller (115.0 ± 27.06) than the overweight/obese PP-BMI group (125.0 ± 25.51).

The mean number of pregnancies for the normal-weight and overweight/obese PP-BMI groups was similar (2.04 ± 0.75 and 2.05 ± 0.83, respectively).

### 4.3. Physical Activity

Data regarding moderate-intensity physical activity was obtained from all study participants (Table 2). Moderate-intensity physical activity was described in the demographic and health survey (Figure S1) questionnaire as an activity increasing breath or heart rate for 10 min.

We observed fewer subjects participating in moderate-intensity fitness or sports activities than just moderate-intensity physical activity. The demographic and health questionnaire (Figure S1) defined moderate-intensity fitness or sports as fitness or sporting activities that increase breath or heart rate for 10 min.

### 4.4. Prenatal Questions

We observed that most of the subjects took prenatal vitamins during their pregnancy (only one subject in the overweight/obese PP-BMI group did not); however, only 15 (63%) subjects in the normal-weight PP-BMI group and 18 (90%) subjects in the overweight/obese PP-BMI group took prenatal vitamins during lactation.

All the subjects were able to provide the type and brand of prenatal vitamins they used, allowing for determination of the DHA quantity they consumed. The DHA content of prenatal vitamins used by the normal and overweight/obese PP-BMI groups were 118.75 ± 105.10 mg and 92.50 ± 92.16 mg, respectively, *p* = 0.39.

HBM samples were collected from mature milk only between two and fourteen weeks (16–104 days) postpartum. HBM samples from normal-weight PP-BMI and overweight/obese PP-BMI groups were collected at 58.33 ± 27.83 and 54.65 ± 32.12 days postpartum, respectively (*p* = 0.69).

### 4.5. Maternal Body Mass Index

Weight and body mass index (BMI) history of the study participants was self-reported (Table 3). All weight and BMI measurements between the normal-weight and the overweight/obese groups were significantly different (*p* < 0.001). The current height between the maternal PP-BMI groups was not significantly different.

### 4.6. Dietary Information

Of the participants who consumed a daily fish or krill oil supplement during pregnancy, six (25%) were from the normal PP-BMI group, and three (15%) were from the overweight/obese PP-BMI group. The number of oil supplements taken by normal-weight and overweight/obese PP-BMI subjects was 2.23 ± 3.11 and 1.38 ± 2.69 supplements per month, respectively (*p* = 0.34). Other participants consumed nuts, including 21 (88%) from the normal PP-BMI group and 19 (95%) from the overweight/obese PP-BMI group, with the average times of consumption at 9.58 ± 9.37 and 11.25 ± 11.32 per month, respectively (*p* = 0.60). We found that fewer individuals consumed flaxseed or flaxseed oil than fish oil and nut consumption, with six (25%) in the normal PP-BMI group and seven (35%) in the overweight/obese PP-BMI group consuming flax regularly. Supplements other than prenatal vitamins were taken by seven (29%) participants in the normal PP-BMI group and four (21%) in the overweight/obese PP-BMI group.

### 4.7. Food Frequency Questionnaires

All study participants completed the BLOCK Fruit and Vegetable Fiber Screener (Figure S2) and the BLOCK Dietary Fat Screener (Figure S3). Consistent across both assessments, no significant differences in dietary intake were detected between the maternal PP-BMI groups. (Table 4).

### 4.8. Proximate Analysis on HBM Samples

HBM macronutrient composition including the total caloric content, protein concentration, fat content and inorganic matter was comparable between the two maternal PP-BMI groups. Across all proximate analysis assays, the HBM from both groups showed similar profiles indicating that maternal BMI did not meaningfully influence the macronutrient content of HBM. See Table 5.

### 4.9. HBM Short- and Long-Chain Fatty Acids

One participant in the normal PP-BMI group was excluded from short- and long-chain fatty analysis due to insufficient sample volume. In the remaining HBM samples subjected to analysis, SCFAs were present at similar levels in both maternal PP-BMI groups. Several SCFAs were low or had undetectable levels in both groups, reflecting their low abundance in HBM. Median levels and corresponding interquartile ranges of SCFAs in HBM of maternal PP-BMI groups are reported in Table 6.

HBM fatty acid profiles including saturated, monosaturated (MUFA), polyunsaturated (PUFA), omega-3, and omega-6 fatty were similar between the maternal PP-BMI groups. A few fatty acids differed between the PP-BMI groups; DHA and EPA were below detection in both maternal PP-BMI groups. Long-chain fatty acid median concentrations and interquartile ranges are presented in Table 7.

### 4.10. Microbial Profiles of HBM from Women of Differing BMIs

In our study of breastfeeding women in the Appalachian Highlands region, we performed HBM microbiome analyses in QIAGEN CLC Genomics Workbench. Of the forty-four HBM samples, thirty-four were subjected to sequence filtering, while two samples were excluded because their read counts were too low (<1000 sequences) compared to the overall sample coverage. Therefore, two samples were excluded from the analyses. All HBM samples were subjected to microbiome analysis and stratified by maternal PP- BMI, normal PP-BMI (*n* = 19), and overweight/obese PP-BMI (*n* = 13).

#### 4.10.1. Relative Abundance

**Figure 1:**
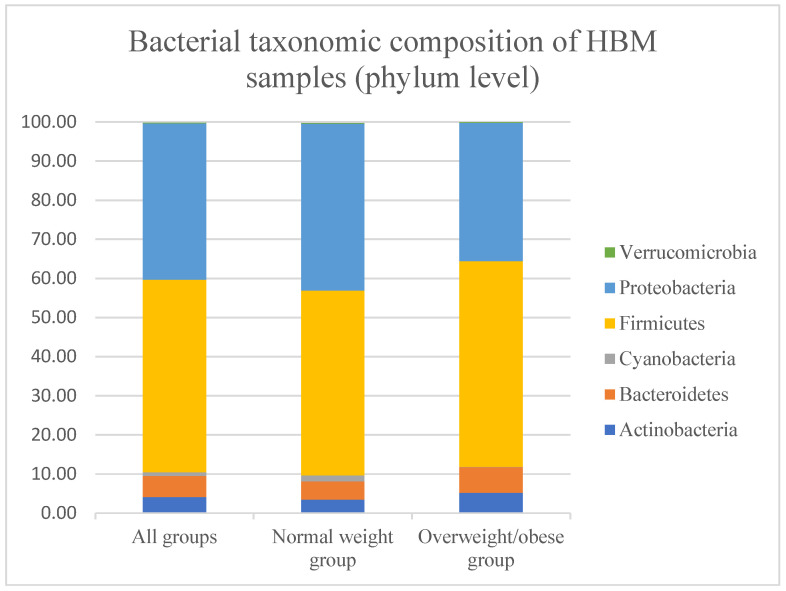
Relative abundance (%) of microbial communities at the phylum level. Each bar represents HBM samples grouped according to maternal PP-BMI status, with coloring indicating the percentage of total reads assigned to each taxon. These stacked bar charts summarize compositional relative abundance data and are used to visualize overall compositional patterns and do not include statistical testing or confidence intervals. Bar charts include all groups (*n* = 32), normal PP-BMI (*n* = 19), and OW/OB PP-BMI (*n* = 13).

The relative abundances of HBM microbiome profiles were compared with mothers of differing BMIs. After quality filtering and trimming of lengths, 1,525,695 16s rRNA sequences were examined with an average number of taxonomically assigned, high-quality sequences of approximately 47,678 sequences per sample. The taxonomic of the sequences showed that the composition of HBM at the phylum level is dominated by Firmicutes at 49.26%, followed by Proteobacteria at 39.97%, Bacteroidetes at 5.35%, and Actinobacteria at 4.11% (Figure 1). Firmicutes, Proteobacteria, Bacteroidetes, and Actinobacteria were the four most predominant bacterial phyla in the groups combined, the normal-weight group, and the overweight/obese group in the same order; however, the abundances in each group differed by group (Table 8).

As in other studies on the HBM microbiome, fourteen phyla were identified in HBM samples: Acidobacteria, Actinobacteria, Armatimonadetes, Bacteroidetes, Chloroflexi, Cyanobacteria, Deferribacteres, Firmicutes, Fusobacteria, Gemmatimonadetes, Planctomycetes, Proteobacteria, Tenericutes, and Verrucomicrobia. The overweight/obese group had no sequences for Acidobacteria, Armatinmonadetes, and Planctomyces, while the normal-weight group had sequences for all the phyla sequenced.

**Figure 2:**
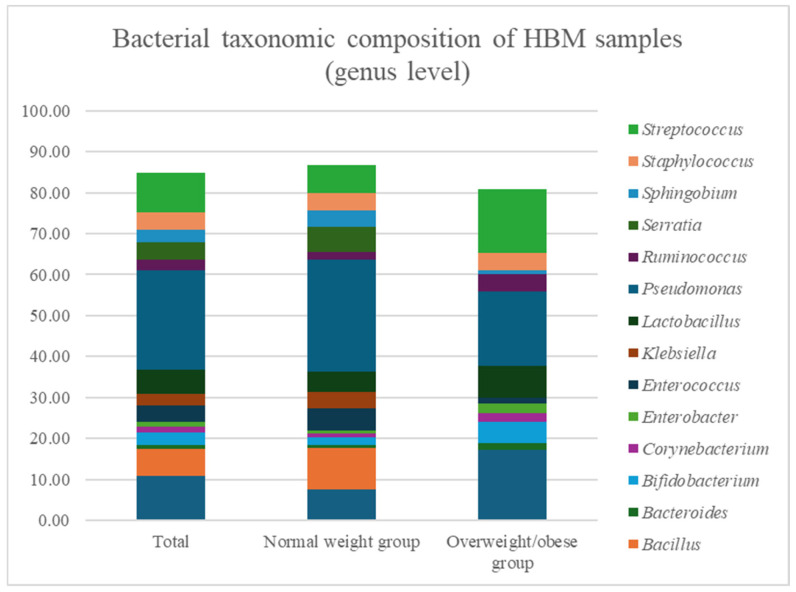
Relative abundance (%) of microbial communities at the genus level. Each bar represents HBM samples grouped according to maternal PP-BMI status, with coloring indicating the percentage of total reads assigned to each taxon. These stacked bar charts summarize compositional relative abundance data and are used to visualize overall compositional patterns and do not include statistical testing or confidence intervals. Bar charts include all groups (*n* = 32), normal PP-BMI (*n* = 19), and OW/OB PP-BMI (*n* = 13).

In the breast milk samples, the most common genera were *Pseudomonas* and *Acinetobacter* (aerobic bacteria from the order of Pseudomonadales), followed by *Streptococcus* (Lactobacillales), *Bacillus* (Bacillales), and *Lactobacillus* (Lactobacillales) (Figure 2). In the normal-weight group HBM, the most common genera were *Pseudomonas* and *Bacillus* followed by *Acinetobacter*, *Streptococcus*, and *Serratia* (Enterobacteriales). The HBM from the overweight/obese group had *Pseudomonas* and *Acinetobacter*, followed by *Streptococcus*, *Lactobacillus*, and *Bifidobacterium* (Bifidobacteriales) (Figure 2). The fifteen most abundant genera identified across all HBM samples are summarized in Table 9. Because stacked bar charts are descriptive visualizations of compositional data, they do not include confidence intervals or statistical testing. Group-level differences were evaluated using GLM-based differential abundance analysis and PERMANOVA.

#### 4.10.2. Alpha Diversity

**Figure 3:**
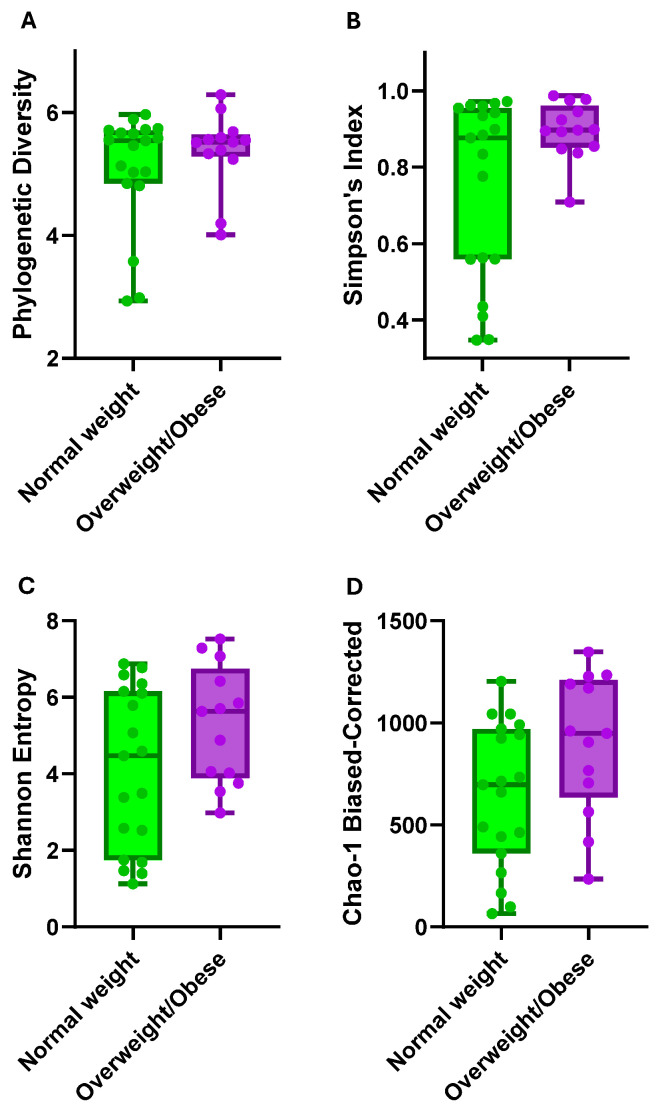
Alpha diversity based on (**A**) phylogenetic diversity, (**B**) Simpson’s index, (**C**) Shannon Entropy, and (**D**) Chao1 Biased Corrected of HBM samples based on maternal PP-BMI with normal weight (*n* = 19) in green and overweight/obese (*n* = 13) in purple.

Alpha diversity is used to measure microbial diversity within a single sample. Alpha diversity measures were calculated using the Kruskal–Wallis test to determine the significance between the medians of the groups of HBM from normal-weight (*n* = 19 in green) and overweight/obese (*n* = 13 in blue) mothers (Figure 3). Phylogenetic differences between species were measured via phylogenetic diversity to determine if normal PP-BMI HBM and overweight/obese PP-BMI HBM significantly differed in biodiversity (Kruskal–Wallis *p* = 0.7) (Figure 3A). Microbial evenness was estimated using Simpson’s index, which was not significant between the maternal groups (Kruskal–Wallis *p* = 0.2) (Figure 3B). Microbial diversity between the groups was calculated using the Shannon index. The Shannon index interprets both bacterial richness and evenness. It has a heightened sensitivity to rare species with species richness, whereas Simpson’s index has a heightened sensitivity to dominant species. No significance was found between normal-weight and overweight/obese PP-BMI HBM groups for Shannon Diversity (Kruskal–Wallis *p* = 0.1) (Figure 3C). No significant differences between the HBM of the normal and overweight/obese PP-BMI groups were found for the Chao1 richness estimator, which calculates microbial richness (Kruskal–Wallis *p* = 0.07) (Figure 3D).

#### 4.10.3. Beta Diversity

**Figure 4:**
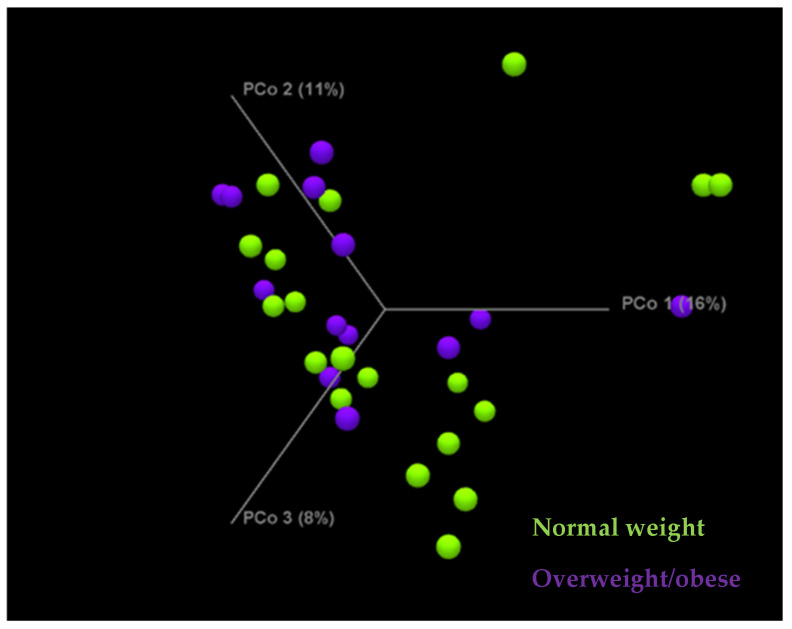
Principal Coordinates of Analysis (PCoA) of beta diversity. A 3D PCoA plot generated by the CLC Microbial Genomics Module displays sample clustering based on Bray–Curtis distances. Axes show the percentage of variance explained by each coordinate. This visualization reflects the default output of the analysis software and is included to maintain reproducibility. Statistical evaluations of group-level differences were assessed separately using PERMANOVA, with R^2^ and *p*-values reported in the Results section. HBM samples are grouped according to maternal PP-BMI with normal weight (*n* = 19) in green and overweight/obese (*n* = 13) in purple.

Bray–Curtis dissimilarity distance measurement was used to measure the similarity or dissimilarity of the two HBM sample groups. Bray–Curtis measures the distances of samples on a Principal Coordinate of Analysis (PCoA) plot and considers the overall abundance (or size) of the sample and the abundance of the taxa (or shape) of the HBM maternal groups. Figure 4 is a three-dimensional PCoA plot showing each sample (circle) and which maternal group it is in (green for normal weight and purple for overweight/obese). Permutational analysis of variance (PERMANOVA) for Bray–Curtis was *p* = 0.18 for beta diversity of the microbiomes of HBM from mothers of differing BMIs. Jaccard, Unweighted UniFrac, and Weighted UniFrac tests of beta diversity were also performed for beta diversity of the microbiomes of HBM from mothers of differing PP-BMIs (*p* = 0.15, *p* = 0.09, *p* = 0.50 respectively).

#### 4.10.4. Differential Abundance

In order to further evaluate similarity or dissimilarity between the maternal PP-BMI groups for the HBM microbiome, an analysis was conducted to determine which OTUs had the most significantly different abundances across all the samples by performing a generalized linear model (GLM) differential abundance test after performing TMM (trimmed mean of M-values) normalization on all samples utilizing the CLC Genomic Workbench Tool for Differential Abundance Analysis.

**Figure 5:**
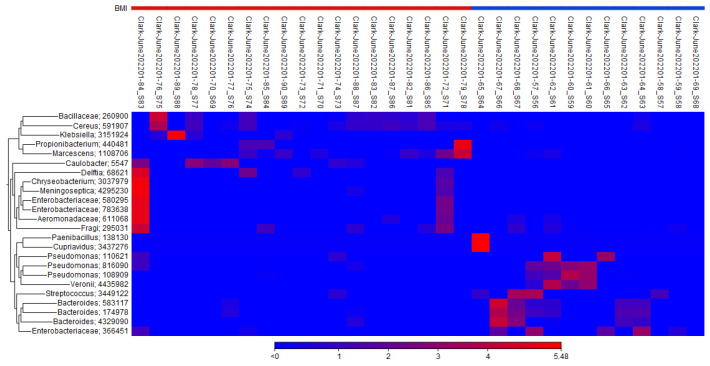
Heatmap of the top 25 differentially abundant taxa between normal-weight and overweight/obese PP-BMI HBM samples. This heatmap displays the top 25 taxa with the greatest differential abundance, with red indicating taxa with the greatest differences between samples, whereas blue indicates taxa with minimal/no difference. The visualization reflects the default output of the CLC Microbial Genomics Module. Differential abundance was assessed using an ANOVA-like GLM with FDR correction, and the full statistical results for these taxa are provided in Table 9. Maternal PP-BMI groups are shown in the bar above the heatmap, with normal weight (*n* = 19) in red and overweight/obese (*n* = 13) in blue.

In the following, the family, genus, and species are denoted as f_x, g_x, or s_x. Firmicutes (f_Bacillaceae g_unknown and f_Bacillaceae, g_*Bacillus*, s_*cereus*), Proteobacteria (f_Enterobacteriaceae, g_*Klebsiella*; f_Enterobacteriaceae, g_*Serratia*, s_*marcescens*; f_Caulobacteraceae, g_*Caulobacter*; f_Comamonadaceae, g_*Delftia*; f_Enterobacteriaceae, g_unknown; f_Aeromonadaceae, g_unknown; f_Pseudomonadaceae, g_*Pseudomonas*, s_*fragi*; and f_Enterobacteriaceae, g_unknown), Bacteroidetes (f_Weeksellaceae, g_*Chryseobacterium*; and f_Weeksellaceae, g_*Elizabethkingia*, s_*meningoseptica*), and Actinobacteria (f_Propionibacteriaceae, g_*Propionibacterium*) were significantly different between normal-weight and overweight/obese PP-BMI HBM samples and had higher relative abundances in HBM samples from normal-weight women compared with overweight/obese women (Figure 5). Firmicutes (f_Paenibacillaceae, g_*Paenibacillus*; and f_Streptococcaceae, g_*Streptococcus*), Proteobacteria (f_Enterobacteriaceae, g_unknown; f_Oxalobacteraceae, g_*Cupriavidus*; f_Pseudomonadaceae, g_*Pseudomonas*; and f_Pseudomonadaceae, g_*Pseudomonas*, s_*veronii*), Bacteroidetes (f_Bacteroidaceae, g_*Bacteroides*) were significantly different between normal-weight and overweight/obese PP-BMI HBM samples and had higher relative abundances in HBM samples from overweight/obese women compared with normal-weight women (Figure 5). Remarkably, even though HBM from overweight/obese mothers had significantly different abundances in taxa, the HBM had low relative abundance levels of bacterial taxa compared to the HBM of normal-weight mothers (Figure 5). To complement the heat map in Figure 5, the top 25 most statistically significant taxa identified by differential abundance analysis are provided in Table 10.

## 5. Discussion

In this study, the distribution of maternal ages was comparable between normal-weight and overweight/obese participants. As expected, PP-BMI was significantly different between the groups, and women who entered adulthood with higher BMI tended to remain in the higher-BMI category at conception. All human breast milk (HBM) samples were collected during the mature milk stage, and the timing of collection was similar across groups.

Patterns of moderate-intensity physical activity were similar and not significantly different between the maternal PP-BMI groups. This similarity may suggest that differences in maternal PP-BMI in this cohort may be more strongly influenced by caloric intake and/or other metabolic factors rather than activity level alone.

Prenatal DHA intake was similar between groups, and most participants reported taking only prenatal vitamins without additional fatty acid supplements. Although fish intake tended to be higher among normal-weight PP-BMI mothers, overall consumption in both groups remained well below recommended levels for pregnant and breastfeeding women [68,69].

Dietary assessments using the BLOCK Fruit and Vegetable Fiber Screener and the BLOCK Dietary Fat Screener indicated no significant differences in fruit and vegetable intake or dietary fat consumption between the maternal PP-BMI groups.

HBM macronutrient composition—including calories, protein, ash (non-organic) and fat—was similar between groups. Although subtle trends were observed, maternal PP-BMI alone may not be a major contributor to macronutrient levels, consistent with findings from other human milk studies. A larger sample size may help clarify whether small group-level differences exist.

Long-chain fatty acid profiles in HBM were also largely comparable between groups, with only a few individual fatty acids differing in relative abundance. Notably, myristic acid tended to be higher in HBM from overweight/obese PP-BMI mothers, whereas stearic acid tended to be higher in HBM from normal PP-BMI mothers. Long-chain omega-3 fatty acids such as EPA and DHA were low across all samples, consistent with the low fish intake reported by participants and the similar DHA content of prenatal vitamins used. This pattern aligns with evidence that EPA and DHA concentrations in HBM reflect maternal fish consumption [68,70,71].

Short-chain fatty acids (SCFAs) in HBM showed a similar pattern between the maternal PP-BMI groups, with most SCFAs present at comparable levels between groups. However, several SCFAs—including butyric, valeric, and isocaproic acids—were more abundant in HBM from normal PP-BMI mothers. Butyrate, one of the major SCFAs, may play a physiological role in intestinal epithelial energy supply, barrier integrity, and anti-inflammatory signaling [72,73,74,75,76], and has been reported to be lower in the breast milk of atopic mothers [77]. The origins of butyrate in HBM remain unclear, with hypotheses suggesting transfer from maternal circulation or in situ production by mammary-associated bacteria [78,79].

Valeric acid has been associated with epithelial growth in the intestine [80,81,82], whereas less is known about the functional relevance of isocaproic acid. It has been proposed as a potential marker of C. difficile activity [83], although no C. difficile sequences were detected in our samples. Both valeric and isocaproic acids can arise from bacterial fermentation of proteins and amino acids [84,85]. The higher protein content observed in HBM from normal PP-BMI mothers may help explain the elevated levels of these SCFAs, although maternal dietary protein intake was not assessed. Additional research is needed to clarify the biological significance of non-major SCFAs in HBM and their potential roles in infant development.

The overall structure of the HBM microbiome was similar between maternal PP-BMI groups, with both groups dominated by Firmicutes, Proteobacteria, Bacteroidetes, and Actinobacteria. Measures of microbial diversity did not differ meaningfully between groups. However, HBM from overweight/obese PP-BMI mothers tended to show lower relative abundance across several bacterial taxa. These patterns may be associated with specific aspects of HBM composition—including fatty acids and microbial communities—with potential downstream effects on infant nutrition and early development.

This study should be interpreted in light of several limitations. Interpretation of the HBM microbiome findings is constrained by maternal and perinatal confounders, including the birth mode, gestational age, antibiotic/medication exposure, time of milk collection, and the absence of a standardized HBM collection procedure. All of these confounders are known to influence HBM microbial composition. Recruitment was conducted through a convenience sample from a Facebook group, resulting in a modest sample size. We purposely limited the cohort to Caucasian women in a single geographic region. These factors may limit the generalizability of the findings to more diverse or geographically varied populations. Additionally, HBM samples were self-collected under real-world, non-observed conditions without standardized procedures for timing, breast side, foremilk/hindmilk status, or pump sterility. As such, the results reflect expressed HBM collected in naturalistic settings rather than strictly standardized HBM composition. Despite these limitations, this study provides valuable insights into how maternal pre-pregnancy BMI may relate to selected HBM fatty acids and microbial patterns, highlighting the need for larger cohorts to further clarify these associations. Findings in this research project cannot distinguish BMI-associated effects from perinatal, dietary manipulation, medication-related, and/or sampling-related confounding.

## 6. Conclusions

Despite being a small-cohort study, this study has several strengths. The study subjects were Caucasian women from the Appalachian Highlands region, allowing us to control for factors that impact maternal HBM components and HBM composition, such as race, ethnicity, and geographical region. Milk collection postpartum was between two and fourteen weeks postpartum, allowing us to control for nutritional and microbial differences previously observed between colostrum, transitional milk, and mature milk. This was a novel study, as SCFAs in HBM has not been studied thoroughly, and the role of SCFAs in HBM and its impact on infant growth and health are yet to be fully known.

We did not obtain birth mode and gestational information from the study subjects. This data on birth and gestation would be essential to assess maternal and infant factors affected by these variables, such as microbiomes and SCFAs. Subjects in this study were from the same region and consumed similar diets, reflected in the food BLOCK Fruit and Vegetable Screener and food BLOCK Fat Screener. The time of day when study subjects collected HBM was not standardized and, therefore, may lead to variations in the composition of nutrients due to known differences in HBM composition due to diurnal rhythms. Additionally, HBM collection was not observed or standardized by the researchers, possibly resulting in variation in how HBM was collected. Finally, the researchers did not have study subjects complete a full food BLOCK questionnaire and therefore did not analyze protein consumption of the maternal groups, which would have been beneficial to determine increased protein concentration and SCFA valeric and isocaproic acids (reflection of bacteria fermenting protein in the gut) in HBM may come from the diet or some other mechanism. Finally, non-significant findings in this research study should also be carefully interpreted as a larger cohort, increased sampling of HBM and a more diverse diet may have results that are different from this study.

### Suggestions for Future Research

The relationship between maternal PP-BMI and HBM components, specifically HBM fatty acid and microbiome composition, should be studied further. Future studies should include a large-scale, diverse study population from different regions. In addition to HBM collection, it would be beneficial to collect both skin and stool microbiome samples from the mother to compare fatty acid and microbiome samples to determine similarities and dissimilarities. Information about variables that may impact microbiome and fatty acid profiles should be collected, including a complete food BLOCK questionnaire, medications, drug use, antibiotic use, and health information, including diabetes status. Lastly, maternal blood should be collected to analyze inflammatory markers.

## Figures and Tables

**Table 1:** Study demographics of subjects grouped by maternal PP-BMI.

	Normal PP-BMI Group (*n* = 24)	OW/OB PP-BMI Group (*n* = 20)
Age (in years)	28.79 (5.04)	28.35 (4.21)
Marital status		
Married	22 (92%)	18 (90%)
Never married	1 (4%)	0 (0%)
Living with a partner	1 (4%)	2 (10%)
Education		
High school equivalent	1 (4%)	4 (20%)
Some college or associate degree	7 (29%)	7 (35%)
Bachelor’s degree or greater	16 (67%)	9 (45%)
Employment status		
Full-time employment	9 (38%)	7 (35%)
Part-time employment	1 (4%)	6 (30%)
Self-employed	1 (4%)	1 (5%)
Full-time homemaker	13 (54%)	4 (20%)
Temporarily unemployed	0 (0%)	2 (10%)
Income		
<$20,000	0 (0%)	3 (15%)
$20,000–$24,999	2 (8%)	2 (10%)
$25,000–$34,999	2 (8%)	2 (10%)
$35,000–$44,999	3 (13%)	2 (10%)
$45,000–$54,999	1 (4%)	2 (10%)
$55,000–$64,999	7 (29%)	1 (5%)
$65,000–$74,999	3 (13%)	2 (10%)
$75,000–$99,999	3 (13%)	4 (20%)
$100,000+	3 (13%)	2 (10%)
Infant birth weight in ounces	115.00 (27.06)	125.00 (25.51)
Number of pregnancies	2.04 (0.75)	2.05 (0.83)

**Table 2:** Moderate-intensity physical activity of maternal PP-BMI groups.

	Normal PP-BMI Group (*n* = 24)	OW/OB PP-BMI Group (*n* = 20)	*p*-Value
Moderate-intensity activity (days per week)	3.38 (2.45)	2.70 (2.23)	*p* = 0.35
Moderate intensity (hours per day)	2.02 (3.02)	2.51 (3.32)	*p* = 0.61
Moderate-intensity fitness or sport (days per week)	1.17 (1.65)	1.63 (1.64)	*p* = 0.36
Moderate-intensity fitness or sport (hours per day)	0.26 (0.37)	0.58 (0.70)	*p* = 0.07

Values expressed as mean (standard deviation). *p*-value significant at α < 0.05.

**Table 3:** Weight and body mass index (BMI) history of maternal groups.

	Normal PP-BMI Group (*n* = 24)	OW/OB PP-BMI Group (*n* = 20)	*p*-Value
Current height (inches)	65.33 (2.24)	64.93 (2.15)	*p* = 0.54
Pre-pregnancy weight (lbs.)	133.71 (12.27)	204.05 (32.87)	*p* < 0.001
Pre-pregnancy BMI (kg/m^2^)	22.03 (1.83)	34.08 (5.66)	*p* < 0.001
Weight at age of 18 (lbs.)	124.08 (16.25)	163.05 (24.84)	*p* < 0.001
BMI at age of 18 years (kg/m^2^)	20.46 (2.62)	27.27 (4.54)	*p* < 0.001
Highest weight when not pregnant (lbs.)	142.00 (15.29)	215.10 (31.31)	*p* < 0.001
Highest BMI when not pregnant (kg/m^2^)	23.39 (2.26)	35.98 (5.78)	*p* < 0.001

Values expressed as mean (standard deviation). *p*-value significant at α < 0.05.

**Table 4:** Results of BLOCK Dietary Fruit–Vegetable–Fiber and Fat Screener for maternal PP-BMI groups.

	Normal PP-BMI Group (*n* = 24)	OW/OB PP-BMI Group (*n* = 20)	*p*-Value
Servings of fruits & vegetables	3.98 (1.62)	4.79 (2.37)	*p* = 0.21
Vitamin C (mg)	137.55 (40.23)	152.65 (59.19)	*p* = 0.34
Magnesium (mg)	342.87 (72.38)	369.80 (104.91)	*p* = 0.34
Potassium (mg)	3290.98 (710.55)	3556.22 (1035.20)	*p* = 0.34
Fiber (g)	16.95 (4.90)	18.76 (7.02)	*p* = 0.34
Cholesterol (mg)	269.13 (49.88)	258.01 (50.43)	*p* = 0.47
Total fat (g)	106.60 (15.35)	103.18 (15.52)	*p* = 0.47
Saturated fat (g)	28.89 (5.63)	27.64 (5.69)	*p* = 0.47
Percent fat (%)	37.78 (3.84)	36.92 (3.88)	*p* = 0.47
Percent saturated fat (%)	10.21 (1.58)	9.86 (1.61)	*p* = 0.47

Values expressed as mean (standard deviation). *p*-value significant at α < 0.05.

**Table 5:** Results of proximate analysis of HBM samples by maternal PP-BMI group.

	Normal PP-BMI Group (*n* = 24)	OW/OB PP-BMI Group (*n* = 20)	*p*-Value
Total Calories (calories/g of DM)	5581.8 (301.3)	5562.9 (403.0)	*p* = 0.86
Percent Protein (%)	9.80 (1.53)	8.93 (2.20)	*p* = 0.13
Percent Fat (%)	39.6 (7.6)	43.9% (9.8)	*p* = 0.11
Percent Inorganics (%)	1.45 (0.44)	1.48 (0.34)	*p* = 0.82

Values expressed as mean (standard deviation). *p*-value significant at *p* < 0.05.

**Table 6:** Short-chain fatty acid analysis results in HBM samples.

Short-Chain Fatty Acid Area Percent							
	Normal PP-BMI Group			OW/OB PP-BMI Group			
	(*n* = 23)			(*n* = 20)			
	Median	25th %ile	75th %ile	Median	25th %ile	75th %ile	*p*-Value
Acetic	33.55	26.46	50.13	44.64	37.84	55.92	0.09
Propionic	0	0	0	0	0	0	0.29
Isobutyric	0	0	1.46	1.34	0	3.76	0.09
Butyric	23.23	12.09	32.22	16.09	11.95	19.61	0.04
Isovaleric	0	0	0	0	0	0	0.67
Valeric	0	0	2.54	0	0	0	0.04
Isocaproic	0	0	0.61	0	0	0	0.02
Caproic	26.94	21.79	30.18	21.27	14.65	26.57	0.11

Wilcoxon rank-sum test. *p*-value significant at *p* = 0.05.

**Table 7:** Long-chain fatty acid analysis results in HBM samples.

Long-Chain Fatty Acid							
	Normal PP-BMI Group (*n* = 23)			OW/OB PP-BMI Group (*n* = 20)			
	Median	25th %ile	75th %ile	Median	25th %ile	75th %ile	*p*-Value
Laurate	3.15	2.20	4.38	4.16	2.66	5.97	0.14
Myristate	4.77	3.33	5.48	6.20	4.18	7.59	0.04
Myristoleate	0.29	0.15	0.42	0.29	0.26	0.33	0.87
Palmitate	20.87	17.28	21.89	21.33	19.30	22.05	0.18
Palmitoleate	3.92	3.15	4.55	3.76	3.55	4.37	0.97
Stearate	19.57	18.65	22.99	17.96	15.89	20.04	0.04
Oleate	21.69	20.20	25.00	22.24	20.13	24.43	0.74
Linoleate	10.31	8.88	16.18	10.47	8.98	11.09	0.48
Alpha Linoleate	11.33	9.12	12.59	11.64	10.17	13.35	0.35
Gamma Linoleate	1.49	1.02	2.43	1.31	1.09	1.86	0.67
Arachidonate	0.27	0.20	0.33	0.20	0.13	0.41	0.42
Eicosapentaenoate	0.00	0.00	0.00	0.00	0.00	0.00	0.38
Docosahexanolate	0.00	0.00	0.09	0.00	0.00	0.00	0.05
Omega 3	1.49	1.17	2.43	1.31	1.09	1.86	0.46
Omega 6	22.45	19.36	26.59	23.14	19.89	25.09	0.78
O3/O6	0.08	0.05	0.08	0.06	0.05	0.07	0.27
O6/O3	13.19	12.03	18.48	16.39	13.36	18.78	0.27
PUFA	23.41	20.58	29.22	24.37	21.06	27.27	0.73
MUFA	26.36	24.12	30.31	26.58	24.03	29.09	0.65
PUFA/MUFA	0.94	0.66	1.20	0.90	0.75	1.05	0.84
MUFA/PUFA	1.06	0.84	1.51	1.11	0.95	1.34	0.84
SAT	48.87	43.98	52.42	50.04	44.64	52.74	0.64
UNSAT	51.13	47.58	56.02	49.96	47.24	54.27	0.54
SAT/UNSAT	0.96	0.79	1.10	1.00	0.84	1.12	0.62
UNSAT/SAT	1.05	0.91	1.27	1.00	0.90	1.19	0.62

Wilcoxon rank-sum test. *p*-value significant at *p* = 0.05.

**Table 8:** The four most abundant phyla found in HBM samples.

	All Groups (*n* = 32)	Normal PP-BMI Group (*n* = 19)	OW/OB PP-BMI Group (*n* = 13)
Firmicutes	49.26	47.29	52.58
Proteobacteria	39.97	42.72	35.35
Bacteroidetes	5.35	4.64	6.55
Actinobacteria	4.11	3.46	5.19

Numbers reported are in percent %.

**Table 9:** The fifteen most abundant genera found in HBM samples.

	All Groups (*n* = 32)	Normal PP-BMI Group (*n* = 19)	OW/OB PP-BMI Group (*n* = 13)
Acinetobacter	10.75	7.61	17.11
Bacillus	6.70	10.01	0.01
Bacteroides	1.04	0.66	1.81
Bifidobacterium	3.00	1.96	5.10
Corynebacterium	1.47	1.09	2.25
Enterobacter	1.11	0.53	2.30
Enterococcus	4.04	5.40	1.29
Klebsiella	2.84	4.20	0.10
Lactobacillus	5.85	4.90	7.77
Pseudomonas	24.32	27.40	18.09
Ruminococcus	2.54	1.74	4.17
Serratia	4.11	6.14	0.01
Sphingobium	3.07	4.01	1.16
Staphylococcus	4.33	4.38	4.23
Streptococcus	9.63	6.73	15.50

Numbers reported are in percent %.

**Table 10:** GLM-based differential abundance statistics for PP-BMI-associated taxa.

Name	Max Group Mean	Log_2_ Fold Change	Fold Change	*p*-Value	FDR *p*-Value	Bonferroni
Paenibacillus; 138130	378.2308	12.01395	4135.801	1.31 × 10^−9^	2.17 × 10^−6^	2.17 × 10^−6^
Pseudomonas; 108909	75.30769	8.763658	434.6342	3.26 × 10^−9^	2.71 × 10^−6^	5.42 × 10^−6^
Bacteroides; 174978	55.07692	7.844084	229.776	1.27 × 10^−8^	7.05 × 10^−6^	2.12 × 10^−5^
Marcescens; 1108706	2169.105	−14.6945	−26,514.1	5.08 × 10^−8^	1.69 × 10^−5^	8.44 × 10^−5^
Veronii; 4435982	77.38462	7.950822	247.4207	4.45 × 10^−8^	1.69 × 10^−5^	7.39 × 10^−5^
Bacteroides; 583117	13.15385	7.197337	146.7622	6.8 × 10^−8^	1.88 × 10^−5^	0.000113
Pseudomonas; 816090	790.5385	9.37183	662.5244	9.26 × 10^−8^	2.2 × 10^−5^	0.000154
Meningoseptica; 4295230	83.78947	−13.131	−8970.38	1.25 × 10^−7^	2.6 × 10^−5^	0.000208
Enterobacteriaceae; 580295	93.89474	−13.0855	−8692.44	1.71 × 10^−7^	2.85 × 10^−5^	0.000285
Bacillaceae; 260900	316.1579	−10.9963	−2042.81	1.59 × 10^−7^	2.85 × 10^−5^	0.000265
Cereus; 591907	3305.263	−10.9794	−2018.91	3.22 × 10^−7^	4.58 × 10^−5^	0.000535
Pseudomonas; 110621	674.5385	8.258151	306.1619	3.31 × 10^−7^	4.58 × 10^−5^	0.00055
Delftia; 68621	17.15789	−10.6221	−1576.08	3.9 × 10^−7^	4.98 × 10^−5^	0.000647
Enterobacteriaceae; 366451	125.2308	7.391351	167.8874	4.82 × 10^−7^	5.73 × 10^−5^	0.000802
Enterobacteriaceae; 783638	31.94737	−11.3572	−2623.4	5.59 × 10^−7^	5.81 × 10^−5^	0.000929
Bacteroides; 4329090	18.15385	6.636103	99.46405	5.3 × 10^−7^	5.81 × 10^−5^	0.00088
Cupriavidus; 3437276	18.30769	7.772178	218.6043	6.57 × 10^−7^	6.42 × 10^−5^	0.001091
Klebsiella; 3151924	20.84211	−10.1168	−1110.33	8.3 × 10^−7^	7.67 × 10^−5^	0.00138
Aeromonadaceae; 611068	76	−10.7648	−1739.94	1.04 × 10^−6^	9.14 × 10^−5^	0.001737
Propionibacterium; 440481	8.789474	−10.1711	−1152.91	1.11 × 10^−6^	9.25 × 10^−5^	0.001851
Caulobacter; 5547	16.15789	−7.93508	−244.736	1.73 × 10^−6^	0.000131	0.002872
Streptococcus; 3449122	50.53846	6.426479	86.01275	1.73 × 10^−6^	0.000131	0.002868
Fragi; 295031	1000.368	−11.5515	−3001.53	2.15 × 10^−6^	0.000149	0.003572
Chryseobacterium; 3037979	9.210526	−9.74003	−855.146	2.08 × 10^−6^	0.000149	0.003458

Data reported as maximum group mean, log_2_ fold change, fold change, and *p*-values (raw, FDR-adjusted, and Bonferroni); differential abundance assessed using ANOVA-like GLM, with significance defined at FDR < 0.05.

## Data Availability

The original contributions presented in this study are included in the article/Supplementary Materials. Further inquiries can be directed to the corresponding author.

## Supplementary Materials

The following supporting information can be downloaded at: https://www.mdpi.com/article/10.3390/nu18121917/s1. Figure S1: Demographic and Health Survey; Figure S2: BLOCK Dietary Fruit & Vegetable Screener; Figure S3: BLOCK Dietary Fat Screener.

## Abbreviations

The following abbreviations are used in this manuscript:

BF3	Boron trifluoride
BMI	Body Mass Index
DHA	Docosahexaenoic acid
DNA	Deoxyribonucleic acid
DPA	Docosapentaenoic acid
EPA	Eicosapentaenoic acid
ETSU	East Tennessee State University
FID	Flame ionizing detector
GLM	Generalized linear model
HCl	Hydrochloric acid
LCFA	Long-chain fatty acids
N	Normal
OTU	Operational taxonomic unit
OWOB	Overweight/obese
PCoA	Principal coordinate of analysis
PCR	Polymerase chain reaction
PERMANOVA	Permutational multivariate analysis of variance
PP-BMI	Pre-pregnancy body mass index
rRNA	Ribosomal ribonucleic acid
SCFA	Short-chain fatty acids
TMM	Trimmed mean of M-values
