# Supplementary material for: Maternal Pre-Pregnancy Body Mass Index and Its Impact on Short- and Long-Chain Fatty Acid and Microbiome Profiles of Human Breast Milk in Caucasian Women of Northeast Tennessee
Source: Nutrients. 2026 Jun 12;18(12):1917. doi: 10.3390/nu18121917 (PMC13304685; doi:10.3390/nu18121917)
Supplement: Supplementary file 1 [file nutrients-18-01917-s001.zip › Descriptives LCFA 05APR26.pdf]

The SAS System

The MEANS Procedure

group	N Obs	Variable	N	Mean	Std Dev	Minimum	25th Pctl	Median	75th Pctl	Maximum
A	23	laurate	23	3.38	1.31	1.52	2.20	3.15	4.38	6.28
		myristate	23	4.77	1.56	2.11	3.33	4.77	5.48	8.94
		myristoleate	23	0.29	0.16	0.06	0.15	0.29	0.42	0.76
		palmitate	23	19.97	2.54	16.30	17.28	20.87	21.89	24.02
		palmitoleate	23	3.95	1.07	2.28	3.15	3.92	4.55	6.08
		stearate	23	20.08	3.26	14.31	18.65	19.57	22.99	26.51
		oleate	23	22.77	3.18	17.50	20.20	21.69	25.00	28.72
		linoleate	23	12.04	4.73	4.82	8.88	10.31	16.18	20.99
		a_Linolenate	23	10.78	3.03	1.95	9.12	11.33	12.59	16.87
		__Linolenate	23	1.64	0.71	0.49	1.02	1.49	2.43	2.90
		arachidonate	23	0.27	0.10	0.11	0.20	0.27	0.33	0.46
		Eicosapentaenoate	23	0.00	0.02	0.00	0.00	0.00	0.00	0.09
		Docosahexanolate	23	0.05	0.08	0.00	0.00	0.00	0.09	0.29
		omega3	23	1.69	0.73	0.49	1.17	1.49	2.43	2.95
		omega6	23	23.08	5.78	12.08	19.36	22.45	26.59	33.64
		o3_o6	23	0.07	0.02	0.04	0.05	0.08	0.08	0.10
		o6_o3	23	15.17	4.42	9.56	12.03	13.19	18.48	24.62
		pufa	23	24.78	6.43	12.57	20.58	23.41	29.22	36.21
		mufa	23	27.01	3.71	20.80	24.12	26.36	30.31	34.60
		pufa_mufa	23	0.95	0.34	0.36	0.66	0.94	1.20	1.65
		mufa_pufa	23	1.20	0.51	0.61	0.84	1.06	1.51	2.75
		sat	23	48.20	4.95	37.30	43.98	48.87	52.42	55.07
		unsat	23	51.78	4.84	44.93	47.58	51.13	56.02	62.71
		sat_unsat	23	0.95	0.18	0.59	0.79	0.96	1.10	1.23
		unsat_sat	23	1.10	0.23	0.82	0.91	1.05	1.27	1.68
B	20	laurate	20	4.18	1.71	1.91	2.66	4.16	5.97	6.54
		myristate	20	6.05	2.01	3.10	4.18	6.20	7.59	10.93
		myristoleate	20	0.29	0.08	0.12	0.26	0.29	0.33	0.47
		palmitate	20	21.27	2.65	17.34	19.30	21.33	22.05	29.19
		palmitoleate	20	3.99	0.63	3.07	3.55	3.76	4.37	5.75
		stearate	20	17.94	2.25	13.62	15.89	17.96	20.04	21.35
		oleate	20	22.01	2.96	16.24	20.13	22.24	24.43	25.75
		linoleate	20	10.75	3.31	5.64	8.98	10.47	11.09	19.25
		a_Linolenate	20	11.51	2.02	6.90	10.17	11.64	13.35	13.93
		__Linolenate	20	1.50	0.58	0.76	1.09	1.31	1.86	2.65
		arachidonate	20	0.27	0.19	0.08	0.13	0.20	0.41	0.71
		Eicosapentaenoate	20	0.00	0.00	0.00	0.00	0.00	0.00	0.00
		Docosahexanolate	20	0.00	0.01	0.00	0.00	0.00	0.00	0.04
		omega3	20	1.51	0.58	0.76	1.09	1.31	1.86	2.65
		omega6	20	22.54	4.09	15.08	19.89	23.14	25.09	30.59
		o3_o6	20	0.07	0.02	0.05	0.05	0.06	0.07	0.10
		o6_o3	20	16.14	3.58	9.99	13.36	16.39	18.78	21.91
		pufa	20	24.05	4.59	16.02	21.06	24.37	27.27	33.02
		mufa	20	26.29	3.20	20.20	24.03	26.58	29.09	31.06
		pufa_mufa	20	0.93	0.23	0.60	0.75	0.90	1.05	1.51
		mufa_pufa	20	1.13	0.26	0.66	0.95	1.11	1.34	1.66
		sat	20	49.44	5.68	39.84	44.64	50.04	52.74	63.20
		unsat	20	50.33	5.54	36.80	47.24	49.96	54.27	60.16
		sat_unsat	20	1.01	0.24	0.66	0.84	1.00	1.12	1.72
		unsat_sat	20	1.04	0.23	0.58	0.90	1.00	1.19	1.51
