# Supplementary material for: Maternal Pre-Pregnancy Body Mass Index and Its Impact on Short- and Long-Chain Fatty Acid and Microbiome Profiles of Human Breast Milk in Caucasian Women of Northeast Tennessee
Source: Nutrients. 2026 Jun 12;18(12):1917. doi: 10.3390/nu18121917 (PMC13304685; doi:10.3390/nu18121917)
Supplement: Supplementary file 1 [file nutrients-18-01917-s001.zip › Descriptives SCFA Percentages 05APR26.pdf]

The SAS System

The MEANS Procedure

group	N Obs	Variable	N	Mean	Std Dev	Minimum	25th Pctl	Median	75th Pctl	Maximum
A	23	acetic	23	39.65	20.24	14.48	26.46	33.55	50.13	87.79
		propionic	23	0.79	3.04	0.00	0.00	0.00	0.00	14.62
		isobuyric	23	1.15	2.19	0.00	0.00	0.00	1.46	7.49
		butyric	23	23.19	12.02	1.92	12.09	23.23	32.22	44.62
		isovaleric	23	0.76	2.57	0.00	0.00	0.00	0.00	11.88
		valeric	23	1.69	3.67	0.00	0.00	0.00	2.54	14.21
		isocaproic	23	0.56	1.26	0.00	0.00	0.00	0.61	5.31
		caproic	23	24.27	9.68	1.89	21.79	26.94	30.18	38.89
		octanoic	23	5.95	3.69	0.00	2.40	6.08	9.65	11.64
B	20	acetic	20	45.58	14.71	13.05	37.84	44.64	55.92	73.26
		propionic	20	0.08	0.29	0.00	0.00	0.00	0.00	1.28
		isobuyric	20	2.32	2.80	0.00	0.00	1.34	3.76	8.96
		butyric	20	16.68	7.20	3.75	11.95	16.09	19.61	33.72
		isovaleric	20	0.43	0.95	0.00	0.00	0.00	0.00	3.38
		valeric	20	0.12	0.52	0.00	0.00	0.00	0.00	2.31
		isocaproic	20	0.00	0.00	0.00	0.00	0.00	0.00	0.00
		caproic	20	21.32	8.58	2.71	14.65	21.27	26.58	39.94
		octanoic	20	9.07	5.58	0.00	4.77	9.39	11.57	21.92
