# Supplementary material for: Maternal Pre-Pregnancy Body Mass Index and Its Impact on Short- and Long-Chain Fatty Acid and Microbiome Profiles of Human Breast Milk in Caucasian Women of Northeast Tennessee
Source: Nutrients. 2026 Jun 12;18(12):1917. doi: 10.3390/nu18121917 (PMC13304685; doi:10.3390/nu18121917)
Supplement: Supplementary file 1 [file nutrients-18-01917-s001.zip › OUTPUT.pdf]

Frequencies

Notes

Output Created		02-APR-2026 14:55:42
Comments		
Input	Data	C: \Users\wahlquist\OneDrive - East Tennessee State University\Andy Clark\SugarMama\Data\Demographics_02APR2026.sav
	Active Dataset	DataSet1
	Filter	<none>
	Weight	<none>
	Split File	<none>
	N of Rows in Working Data File	44
Missing Value Handling	Definition of Missing	User-defined missing values are treated as missing.
	Cases Used	Statistics are based on all cases with valid data.
Syntax		FREQUENCIES VARIABLES=Group /ORDER=ANALYSIS.
Resources	Processor Time	00:00:00.00
	Elapsed Time	00:00:00.00

Statistics

Group

N	Valid	44
	Missing	0

Group

		Frequency	Percent	Valid Percent	Cumulative Percent
Valid	A	24	54.5	54.5	54.5
	B	20	45.5	45.5	100.0
	Total	44	100.0	100.0	

Means

Notes

Output Created		02-APR-2026 15:17:51
Comments		
Input	Data	C:\Users\wahlquist\OneDrive - East Tennessee State University\Andy Clark\SugarMama\Data\Demographics_02APR2026.sav
	Active Dataset	DataSet1
	Filter	<none>
	Weight	<none>
	Split File	<none>
	N of Rows in Working Data File	44
Missing Value Handling	Definition of Missing	For each dependent variable in a table, user-defined missing values for the dependent and all grouping variables are treated as missing.
	Cases Used	Cases used for each table have no missing values in any independent variable, and not all dependent variables have missing values.
Syntax		MEANS TABLES=age BY Group /CELLS=MEAN COUNT STDDEV.
Resources	Processor Time	00:00:00.00
	Elapsed Time	00:00:00.00

Case Processing Summary

	Cases					
	Included		Excluded		Total	
	N	Percent	N	Percent	N	Percent
age * Group	44	100.0%	0	0.0%	44	100.0%

Report

age

Group	Mean	N	Std. Deviation
A	28.79	24	5.039
B	28.35	20	4.209
Total	28.59	44	4.632

Crosstabs

Notes

Output Created		02-APR-2026 15:18:18
Comments		
Input	Data	C: \Users\wahlquist\OneDrive - East Tennessee State University\Andy Clark\SugarMama\Data\Demographics_02APR2026.sav
	Active Dataset	DataSet1
	Filter	<none>
	Weight	<none>
	Split File	<none>
	N of Rows in Working Data File	44
Missing Value Handling	Definition of Missing	User-defined missing values are treated as missing.
	Cases Used	Statistics for each table are based on all the cases with valid data in the specified range(s) for all variables in each table.
Syntax		CROSSTABS /TABLES=marital education employment BY Group /FORMAT=AVALUE TABLES /CELLS=COUNT COLUMN /COUNT ROUND CELL.
Resources	Processor Time	00:00:00.00
	Elapsed Time	00:00:00.00
	Dimensions Requested	2
	Cells Available	524245

Case Processing Summary

	Cases					
	Valid		Missing		Total	
	N	Percent	N	Percent	N	Percent
marital * Group	44	100.0%	0	0.0%	44	100.0%
education * Group	44	100.0%	0	0.0%	44	100.0%
employment * Group	44	100.0%	0	0.0%	44	100.0%

marital * Group Crosstabulation

			Group		Total
			A	B	
marital	Married	Count	22	18	40
		% within Group	91.7%	90.0%	90.9%
	Never Married	Count	1	0	1
		% within Group	4.2%	0.0%	2.3%
	Living with Partner	Count	1	2	3
		% within Group	4.2%	10.0%	6.8%
Total		Count	24	20	44
		% within Group	100.0%	100.0%	100.0%

education * Group Crosstabulation

			Group		Total
			A	B	
education	High school graduate/GED or equivalent	Count	1	4	5
		% within Group	4.2%	20.0%	11.4%
	Some college or AA degree	Count	7	7	14
		% within Group	29.2%	35.0%	31.8%
	College graduate or above	Count	16	9	25
		% within Group	66.7%	45.0%	56.8%
Total		Count	24	20	44
		% within Group	100.0%	100.0%	100.0%

employment * Group Crosstabulation

			Group		
			A	B	Total
employment	Work for someone else full time	Count	9	7	16
		% within Group	37.5%	35.0%	36.4%
	Temporarily unemployed	Count	0	2	2
		% within Group	0.0%	10.0%	4.5%
	Self-employed	Count	1	1	2
		% within Group	4.2%	5.0%	4.5%
	Work for someone else part time	Count	1	6	7
		% within Group	4.2%	30.0%	15.9%
	Full time homemaker	Count	13	4	17
		% within Group	54.2%	20.0%	38.6%
Total		Count	24	20	44
		% within Group	100.0%	100.0%	100.0%

Crosstabs

Notes

Output Created		02-APR-2026 15:29:45
Comments		
Input	Data	C:\Users\wahlquist\OneDrive - East Tennessee State University\Andy Clark\SugarMama\Data\Demographics_02APR2026.sav
	Active Dataset	DataSet1
	Filter	<none>
	Weight	<none>
	Split File	<none>
	N of Rows in Working Data File	44
Missing Value Handling	Definition of Missing	User-defined missing values are treated as missing.
	Cases Used	Statistics for each table are based on all the cases with valid data in the specified range(s) for all variables in each table.

Notes

Syntax		CROSSTABS<br>/TABLES=income2 BY Group<br>/FORMAT=AVALUE TABLES<br>/CELLS=COUNT COLUMN<br>/COUNT ROUND CELL.
Resources	Processor Time	00:00:00.00
	Elapsed Time	00:00:00.00
	Dimensions Requested	2
	Cells Available	524245

Case Processing Summary

	Cases					
	Valid		Missing		Total	
	N	Percent	N	Percent	N	Percent
income2 * Group	44	100.0%	0	0.0%	44	100.0%

income2 * Group Crosstabulation

			Group		
			A	B	Total
income2	<$20,000	Count	0	3	3
		% within Group	0.0%	15.0%	6.8%
	$20,000-$24,999	Count	2	2	4
		% within Group	8.3%	10.0%	9.1%
	$25,000-$34,999	Count	2	2	4
		% within Group	8.3%	10.0%	9.1%
	$35,000-$44,999	Count	3	2	5
		% within Group	12.5%	10.0%	11.4%
	$45,000-$54,999	Count	1	2	3
		% within Group	4.2%	10.0%	6.8%
	$55,000-$64,999	Count	7	1	8
		% within Group	29.2%	5.0%	18.2%
	$65,000-$74,999	Count	3	2	5
		% within Group	12.5%	10.0%	11.4%
	$75,000-$99,999	Count	3	4	7
		% within Group	12.5%	20.0%	15.9%

income2 * Group Crosstabulation

		Group		Total
		A	B	
$100,000+	Count	3	2	5
	% within Group	12.5%	10.0%	11.4%
Total	Count	24	20	44
	% within Group	100.0%	100.0%	100.0%

T-Test

Notes

Output Created		02-APR-2026 15:34:11
Comments		
Input	Data	C:\Users\wahlquist\OneDrive - East Tennessee State University\Andy Clark\SugarMama\Data\Demographics_02APR2026.sav
	Active Dataset	DataSet1
	Filter	<none>
	Weight	<none>
	Split File	<none>
	N of Rows in Working Data File	44
Missing Value Handling	Definition of Missing	User defined missing values are treated as missing.
	Cases Used	Statistics for each analysis are based on the cases with no missing or out-of-range data for any variable in the analysis.
Syntax		T-TEST GROUPS=Group ('A' 'B')<br>/MISSING=ANALYSIS<br>/VARIABLES=days_pa hours_pa days_sports hours_sports<br>/ES DISPLAY(TRUE)<br>/CRITERIA=CI(.95).
Resources	Processor Time	00:00:00.00
	Elapsed Time	00:00:00.00

Group Statistics

	Group	N	Mean	Std. Deviation	Std. Error Mean
days_pa	A	24	3.38	2.446	.499
	B	20	2.70	2.227	.498
hours_pa	A	24	2.0208	3.01709	.61586
	B	20	2.5125	3.31710	.74173
days_sports	A	24	1.167	1.6528	.3374
	B	20	1.625	1.6374	.3661
hours_sports	A	24	.26042	.372121	.075959
	B	20	.58125	.695705	.155564

Independent Samples Test

		Levene's Test for Equality of Variances		t-test for Equality of Means	
		F	Sig.	t	df
days_pa	Equal variances assumed	.220	.642	.949	42
	Equal variances not assumed			.957	41.641
hours_pa	Equal variances assumed	.886	.352	-.514	42
	Equal variances not assumed			-.510	38.939
days_sports	Equal variances assumed	.000	.984	-.920	42
	Equal variances not assumed			-.921	40.716
hours_sports	Equal variances assumed	7.972	.007	-1.952	42
	Equal variances not assumed			-1.853	27.833

Independent Samples Test

		t-test for Equality of Means			
		Significance			
		One-Sided p	Two-Sided p	Mean Difference	Std. Error Difference
days_pa	Equal variances assumed	.174	.348	.675	.711
	Equal variances not assumed	.172	.344	.675	.705
hours_pa	Equal variances assumed	.305	.610	-.49167	.95563
	Equal variances not assumed	.306	.613	-.49167	.96407
days_sports	Equal variances assumed	.181	.363	-.4583	.4983
	Equal variances not assumed	.181	.363	-.4583	.4979
hours_sports	Equal variances assumed	.029	.058	-.320833	.164384
	Equal variances not assumed	.037	.074	-.320833	.173118

Independent Samples Test

		t-test for Equality of Means	
		95% Confidence Interval of the Difference	
		Lower	Upper
days_pa	Equal variances assumed	-.760	2.110
	Equal variances not assumed	-.748	2.098
hours_pa	Equal variances assumed	-2.42020	1.43687
	Equal variances not assumed	-2.44179	1.45846
days_sports	Equal variances assumed	-1.4640	.5473
	Equal variances not assumed	-1.4640	.5473
hours_sports	Equal variances assumed	-.652573	.010906
	Equal variances not assumed	-.675546	.033880

Independent Samples Effect Sizes

		Standardizer[a]	Point Estimate	95% Confidence Interval	
				Lower	Upper
days_pa	Cohen's d	2.349	.287	-.311	.882
	Hedges' correction	2.392	.282	-.305	.866
	Glass's delta	2.227	.303	-.302	.900
hours_pa	Cohen's d	3.15634	-.156	-.749	.439
	Hedges' correction	3.21414	-.153	-.736	.432
	Glass's delta	3.31710	-.148	-.742	.449
days_sports	Cohen's d	1.6459	-.278	-.873	.320
	Hedges' correction	1.6760	-.273	-.858	.314
	Glass's delta	1.6374	-.280	-.876	.324
hours_sports	Cohen's d	.542941	-.591	-1.194	.019
	Hedges' correction	.552883	-.580	-1.173	.019
	Glass's delta	.695705	-.461	-1.066	.156

a. The denominator used in estimating the effect sizes.
Cohen's d uses the pooled standard deviation.
Hedges' correction uses the pooled standard deviation, plus a correction factor.
Glass's delta uses the sample standard deviation of the control (i.e., the second) group.

T-Test

Notes

Output Created		02-APR-2026 15:43:44
Comments		
Input	Data	C:\Users\wahlquist\OneDrive - East Tennessee State University\Andy Clark\SugarMama\Data\Demographics_02APR2026.sav
	Active Dataset	DataSet1
	Filter	<none>
	Weight	<none>
	Split File	<none>
	N of Rows in Working Data File	44
Missing Value Handling	Definition of Missing	User defined missing values are treated as missing.
	Cases Used	Statistics for each analysis are based on the cases with no missing or out-of-range data for any variable in the analysis.
Syntax		T-TEST GROUPS=Group ('A' 'B')<br>/MISSING=ANALYSIS<br><br>/VARIABLES=baby_bw_oz<br>/ES DISPLAY(TRUE)...
Resources	Processor Time	00:00:00.00
	Elapsed Time	00:00:00.00

Group Statistics

	Group	N	Mean	Std. Deviation	Std. Error Mean
baby_bw_oz	A	24	115.0000	27.05550	5.52268
	B	20	125.0000	25.50542	5.70318

Independent Samples Test

		Levene's Test for Equality of Variances		t-test for Equality of Means	
		F	Sig.	t	df
baby_bw_oz	Equal variances assumed	.180	.673	-1.253	42
	Equal variances not assumed			-1.260	41.323

Independent Samples Test

		t-test for Equality of Means			
		Significance			
		One-Sided p	Two-Sided p	Mean Difference	Std. Error Difference
baby_bw_oz	Equal variances assumed	.109	.217	-10.00000	7.98257
	Equal variances not assumed	.107	.215	-10.00000	7.93891

Independent Samples Test

		t-test for Equality of Means	
		95% Confidence Interval of the Difference	
		Lower	Upper
baby_bw_oz	Equal variances assumed	-26.10948	6.10948
	Equal variances not assumed	-26.02915	6.02915

Independent Samples Effect Sizes

		Standardizer[a]	Point Estimate	95% Confidence Interval	
				Lower	Upper
baby_bw_oz	Cohen's d	26.36556	-.379	-.976	.222
	Hedges' correction	26.84835	-.372	-.958	.218
	Glass's delta	25.50542	-.392	-.993	.219

a. The denominator used in estimating the effect sizes.
Cohen's d uses the pooled standard deviation.
Hedges' correction uses the pooled standard deviation, plus a correction factor.
Glass's delta uses the sample standard deviation of the control (i.e., the second) group.

Crosstabs

Notes

Output Created		02-APR-2026 15:47:18
Comments		
Input	Data	C:\Users\wahlquist\OneDrive - East Tennessee State University\Andy Clark\SugarMama\Data\Demographics_02APR2026.sav
	Active Dataset	DataSet1
	Filter	<none>
	Weight	<none>
	Split File	<none>
	N of Rows in Working Data File	44
Missing Value Handling	Definition of Missing	User-defined missing values are treated as missing.
	Cases Used	Statistics for each table are based on all the cases with valid data in the specified range(s) for all variables in each table.
Syntax		CROSSTABS<br><br>/TABLES=prenatal_vitamin current_prenatal_vitamin BY Group<br>/FORMAT=AVALUE TABLES<br>/CELLS=COUNT COLUMN<br>/COUNT ROUND CELL.
Resources	Processor Time	00:00:00.00
	Elapsed Time	00:00:00.01
	Dimensions Requested	2
	Cells Available	524245

Case Processing Summary

	Cases					
	Valid		Missing		Total	
	N	Percent	N	Percent	N	Percent
prenatal_vitamin * Group	44	100.0%	0	0.0%	44	100.0%
current_prenatal_vitamin * Group	44	100.0%	0	0.0%	44	100.0%

prenatal_vitamin * Group Crosstabulation

			Group		Total
			A	B	
prenatal_vitamin	Yes	Count	24	19	43
		% within Group	100.0%	95.0%	97.7%
	No	Count	0	1	1
		% within Group	0.0%	5.0%	2.3%
Total		Count	24	20	44
		% within Group	100.0%	100.0%	100.0%

current_prenatal_vitamin * Group Crosstabulation

			Group		Total
			A	B	
current_prenatal_vitamin	Yes	Count	15	18	33
		% within Group	62.5%	90.0%	75.0%
	No	Count	9	2	11
		% within Group	37.5%	10.0%	25.0%
Total		Count	24	20	44
		% within Group	100.0%	100.0%	100.0%

T-Test

Notes

Output Created		02-APR-2026 15:50:01
Comments		
Input	Data	C:\Users\wahlquist\OneDrive - East Tennessee State University\Andy Clark\SugarMama\Data\Demographics_02APR2026.sav
	Active Dataset	DataSet1
	Filter	<none>
	Weight	<none>
	Split File	<none>
	N of Rows in Working Data File	44
Missing Value Handling	Definition of Missing	User defined missing values are treated as missing.
	Cases Used	Statistics for each analysis are based on the cases with no missing or out-of-range data for any variable in the analysis.
Syntax		T-TEST GROUPS=Group('A' 'B')<br>/MISSING=ANALYSIS<br>/VARIABLES=num_pregnancy<br>/ES DISPLAY(TRUE)<br>/CRITERIA=CI(.95).
Resources	Processor Time	00:00:00.00
	Elapsed Time	00:00:00.00

Group Statistics

	Group	N	Mean	Std. Deviation	Std. Error Mean
num_pregnancy	A	24	2.04	.751	.153
	B	20	2.05	.826	.185

Independent Samples Test

		Levene's Test for Equality of Variances		t-test for Equality of Means	
		F	Sig.	t	df
num_pregnancy	Equal variances assumed	.010	.921	-.035	42
	Equal variances not assumed			-.035	38.931

Independent Samples Test

		t-test for Equality of Means		
		Significance		
		One-Sided p	Two-Sided p	Mean Difference
num_pregnancy	Equal variances assumed	.486	.972	-.008
	Equal variances not assumed	.486	.972	-.008

Independent Samples Test

		t-test for Equality of Means		
		Std. Error Difference	95% Confidence Interval of the Difference	
			Lower	Upper
num_pregnancy	Equal variances assumed	.238	-.488	.472
	Equal variances not assumed	.240	-.494	.477

Independent Samples Effect Sizes

		Standardizer[a]	Point Estimate	95% Confidence Interval	
				Lower	Upper
num_pregnancy	Cohen's d	.785	-.011	-.604	.583
	Hedges' correction	.800	-.010	-.593	.572
	Glass's delta	.826	-.010	-.603	.583

a. The denominator used in estimating the effect sizes.
Cohen's d uses the pooled standard deviation.
Hedges' correction uses the pooled standard deviation, plus a correction factor.
Glass's delta uses the sample standard deviation of the control (i.e., the second) group.

Crosstabs

Notes

Output Created		02-APR-2026 15:52:22
Comments		
Input	Data	C: \Users\wahlquist\OneDrive - East Tennessee State University\Andy Clark\SugarMama\Data\Demographics_02APR2026.sav
	Active Dataset	DataSet1
	Filter	<none>
	Weight	<none>
	Split File	<none>
	N of Rows in Working Data File	44
Missing Value Handling	Definition of Missing	User-defined missing values are treated as missing.
	Cases Used	Statistics for each table are based on all the cases with valid data in the specified range(s) for all variables in each table.
Syntax		CROSSTABS<br>/TABLES=baby_sex BY Group<br>/FORMAT=AVALUE TABLES<br>/CELLS=COUNT COLUMN<br>/COUNT ROUND CELL.
Resources	Processor Time	00:00:00.00
	Elapsed Time	00:00:00.01
	Dimensions Requested	2
	Cells Available	524245

Case Processing Summary

	Cases					
	Valid		Missing		Total	
	N	Percent	N	Percent	N	Percent
baby_sex * Group	44	100.0%	0	0.0%	44	100.0%

baby_sex * Group Crosstabulation

			Group		
			A	B	Total
baby_sex	1	Count	13	8	21
		% within Group	54.2%	40.0%	47.7%
	1, 1, 2	Count	1	0	1
		% within Group	4.2%	0.0%	2.3%
	1, 2	Count	0	1	1
		% within Group	0.0%	5.0%	2.3%
	2	Count	10	11	21
		% within Group	41.7%	55.0%	47.7%
Total		Count	24	20	44
		% within Group	100.0%	100.0%	100.0%

T-Test

Notes

Output Created		02-APR-2026 15:54:41
Comments		
Input	Data	C:\Users\wahlquist\OneDrive - East Tennessee State University\Andy Clark\SugarMama\Data\Demographics_02APR2026.sav
	Active Dataset	DataSet1
	Filter	<none>
	Weight	<none>
	Split File	<none>
	N of Rows in Working Data File	44
Missing Value Handling	Definition of Missing	User defined missing values are treated as missing.
	Cases Used	Statistics for each analysis are based on the cases with no missing or out-of-range data for any variable in the analysis.
Syntax		T-TEST GROUPS=Group('A' 'B')<br>/MISSING=ANALYSIS<br>/VARIABLES=DHA<br>/ES DISPLAY(TRUE)<br>/CRITERIA=CI(.95).

Notes

Resources	Processor Time	00:00:00.00
	Elapsed Time	00:00:00.00

Group Statistics

	Group	N	Mean	Std. Deviation	Std. Error Mean
DHA	A	24	118.75	105.101	21.454
	B	20	92.50	92.160	20.608

Independent Samples Test

		Levene's Test for Equality of Variances		t-test for Equality of Means	
		F	Sig.	t	df
DHA	Equal variances assumed	1.739	.194	.872	42
	Equal variances not assumed			.882	41.872

Independent Samples Test

		t-test for Equality of Means			
		Significance			
		One-Sided p	Two-Sided p	Mean Difference	Std. Error Difference
DHA	Equal variances assumed	.194	.388	26.250	30.112
	Equal variances not assumed	.191	.383	26.250	29.748

Independent Samples Test

		t-test for Equality of Means	
		95% Confidence Interval of the Difference	
		Lower	Upper
DHA	Equal variances assumed	-34.518	87.018
	Equal variances not assumed	-33.789	86.289

Independent Samples Effect Sizes

		Standardizer[a]	Point Estimate	95% Confidence Interval	
				Lower	Upper
DHA	Cohen's d	99.455	.264	-.334	.858
	Hedges' correction	101.277	.259	-.328	.843
	Glass's delta	92.160	.285	-.319	.881

a. The denominator used in estimating the effect sizes.
Cohen's d uses the pooled standard deviation.
Hedges' correction uses the pooled standard deviation, plus a correction factor.
Glass's delta uses the sample standard deviation of the control (i.e., the second) group.

Crosstabs

Notes

Output Created		02-APR-2026 16:00:16
Comments		
Input	Data	C:\Users\wahlquist\OneDrive - East Tennessee State University\Andy Clark\SugarMama\Data\Demographics_02APR2026.sav
	Active Dataset	DataSet1
	Filter	<none>
	Weight	<none>
	Split File	<none>
	N of Rows in Working Data File	44
Missing Value Handling	Definition of Missing	User-defined missing values are treated as missing.
	Cases Used	Statistics for each table are based on all the cases with valid data in the specified range(s) for all variables in each table.

Notes

Syntax		CROSSTABS<br><br>/TABLES=lactationsample_day lactationsample_wk lactation_sample_rounded_wk BY Group<br>/FORMAT=AVALUE TABLES<br>/CELLS=COUNT COLUMN<br>/COUNT ROUND CELL.
Resources	Processor Time	00:00:00.00
	Elapsed Time	00:00:00.01
	Dimensions Requested	2
	Cells Available	524245

Case Processing Summary

	Cases					
	Valid		Missing		Total	
	N	Percent	N	Percent	N	Percent
lactationsample_day * Group	44	100.0%	0	0.0%	44	100.0%
lactationsample_wk * Group	44	100.0%	0	0.0%	44	100.0%
lactation_sample_rounded_wk * Group	44	100.0%	0	0.0%	44	100.0%

lactationsample_day * Group Crosstabulation

			Group		Total
			A	B	
lactationsample_day	16	Count	0	2	2
		% within Group	0.0%	10.0%	4.5%
	17	Count	1	1	2
		% within Group	4.2%	5.0%	4.5%
	18	Count	0	1	1
		% within Group	0.0%	5.0%	2.3%
	21	Count	0	2	2
		% within Group	0.0%	10.0%	4.5%
	25	Count	0	1	1
		% within Group	0.0%	5.0%	2.3%
	26	Count	1	0	1
		% within Group	4.2%	0.0%	2.3%
	30	Count	1	0	1
		% within Group	4.2%	0.0%	2.3%
	33	Count	2	0	2
		% within Group	8.3%	0.0%	4.5%
	35	Count	1	0	1
		% within Group	4.2%	0.0%	2.3%
	37	Count	1	0	1
		% within Group	4.2%	0.0%	2.3%
	39	Count	1	1	2
		% within Group	4.2%	5.0%	4.5%
	41	Count	0	1	1
		% within Group	0.0%	5.0%	2.3%
	42	Count	1	0	1
		% within Group	4.2%	0.0%	2.3%
	45	Count	1	0	1
		% within Group	4.2%	0.0%	2.3%
	47	Count	2	0	2
		% within Group	8.3%	0.0%	4.5%
	49	Count	2	0	2
		% within Group	8.3%	0.0%	4.5%
	54	Count	0	1	1
		% within Group	0.0%	5.0%	2.3%
	56	Count	0	1	1
		% within Group	0.0%	5.0%	2.3%
	57	Count	1	0	1
		% within Group	4.2%	0.0%	2.3%

lactationsample_day * Group Crosstabulation

			Group		
			A	B	Total
	61	Count	1	0	1
		% within Group	4.2%	0.0%	2.3%
	63	Count	0	2	2
		% within Group	0.0%	10.0%	4.5%
	82	Count	0	1	1
		% within Group	0.0%	5.0%	2.3%
	83	Count	1	0	1
		% within Group	4.2%	0.0%	2.3%
	84	Count	1	2	3
		% within Group	4.2%	10.0%	6.8%
	87	Count	1	0	1
		% within Group	4.2%	0.0%	2.3%
	93	Count	0	1	1
		% within Group	0.0%	5.0%	2.3%
	97	Count	1	0	1
		% within Group	4.2%	0.0%	2.3%
	98	Count	1	2	3
		% within Group	4.2%	10.0%	6.8%
	99	Count	1	0	1
		% within Group	4.2%	0.0%	2.3%
	101	Count	1	0	1
		% within Group	4.2%	0.0%	2.3%
	104	Count	1	1	2
		% within Group	4.2%	5.0%	4.5%
Total		Count	24	20	44
		% within Group	100.0%	100.0%	100.0%

lactationsample_wk * Group Crosstabulation

			Group		
			A	B	Total
lactationsample_wk	2.29	Count	0	2	2
		% within Group	0.0%	10.0%	4.5%
	2.43	Count	1	1	2
		% within Group	4.2%	5.0%	4.5%
	2.57	Count	0	1	1
		% within Group	0.0%	5.0%	2.3%
	3.00	Count	0	2	2
		% within Group	0.0%	10.0%	4.5%
	3.57	Count	0	1	1
		% within Group	0.0%	5.0%	2.3%
	3.71	Count	1	0	1
		% within Group	4.2%	0.0%	2.3%
	4.29	Count	1	0	1
		% within Group	4.2%	0.0%	2.3%
	4.71	Count	2	0	2
		% within Group	8.3%	0.0%	4.5%
	5.00	Count	1	0	1
		% within Group	4.2%	0.0%	2.3%
	5.29	Count	1	0	1
		% within Group	4.2%	0.0%	2.3%
	5.57	Count	1	1	2
		% within Group	4.2%	5.0%	4.5%
	5.86	Count	0	1	1
		% within Group	0.0%	5.0%	2.3%
	6.00	Count	1	0	1
		% within Group	4.2%	0.0%	2.3%
	6.43	Count	1	0	1
		% within Group	4.2%	0.0%	2.3%
	6.71	Count	2	0	2
		% within Group	8.3%	0.0%	4.5%
	7.00	Count	2	0	2
		% within Group	8.3%	0.0%	4.5%
	7.71	Count	0	1	1
		% within Group	0.0%	5.0%	2.3%
	8.00	Count	0	1	1
		% within Group	0.0%	5.0%	2.3%
	8.14	Count	1	0	1
		% within Group	4.2%	0.0%	2.3%

lactationsample_wk * Group Crosstabulation

			Group		Total
			A	B	
	8.71	Count	1	0	1
		% within Group	4.2%	0.0%	2.3%
	9.00	Count	0	2	2
		% within Group	0.0%	10.0%	4.5%
	11.71	Count	0	1	1
		% within Group	0.0%	5.0%	2.3%
	11.86	Count	1	0	1
		% within Group	4.2%	0.0%	2.3%
	12.00	Count	1	2	3
		% within Group	4.2%	10.0%	6.8%
	12.43	Count	1	0	1
		% within Group	4.2%	0.0%	2.3%
	13.29	Count	0	1	1
		% within Group	0.0%	5.0%	2.3%
	13.86	Count	1	0	1
		% within Group	4.2%	0.0%	2.3%
	14.00	Count	1	2	3
		% within Group	4.2%	10.0%	6.8%
	14.14	Count	1	0	1
		% within Group	4.2%	0.0%	2.3%
	14.43	Count	1	0	1
		% within Group	4.2%	0.0%	2.3%
	14.86	Count	1	1	2
		% within Group	4.2%	5.0%	4.5%
Total		Count	24	20	44
		% within Group	100.0%	100.0%	100.0%

lactation_sample_rounded_wk * Group Crosstabulation

			Group		Total
			A	B	
lactation_sample_rounded_wk	2.00	Count	1	4	5
		% within Group	4.2%	20.0%	11.4%
	3.00	Count	1	3	4
		% within Group	4.2%	15.0%	9.1%
	4.00	Count	3	0	3
		% within Group	12.5%	0.0%	6.8%
	5.00	Count	3	2	5
		% within Group	12.5%	10.0%	11.4%
	6.00	Count	4	0	4
		% within Group	16.7%	0.0%	9.1%
	7.00	Count	2	1	3
		% within Group	8.3%	5.0%	6.8%
	8.00	Count	2	1	3
		% within Group	8.3%	5.0%	6.8%
	9.00	Count	0	2	2
		% within Group	0.0%	10.0%	4.5%
	11.00	Count	1	1	2
		% within Group	4.2%	5.0%	4.5%
	12.00	Count	2	2	4
		% within Group	8.3%	10.0%	9.1%
	13.00	Count	1	1	2
		% within Group	4.2%	5.0%	4.5%
	14.00	Count	4	3	7
		% within Group	16.7%	15.0%	15.9%
Total		Count	24	20	44
		% within Group	100.0%	100.0%	100.0%

T-Test

Notes

Output Created		02-APR-2026 16:01:29
Comments		
Input	Data	C:\Users\wahlquist\OneDrive - East Tennessee State University\Andy Clark\SugarMama\Data\Demographics_02APR2026.sav
	Active Dataset	DataSet1
	Filter	<none>
	Weight	<none>
	Split File	<none>
	N of Rows in Working Data File	44
Missing Value Handling	Definition of Missing	User defined missing values are treated as missing.
	Cases Used	Statistics for each analysis are based on the cases with no missing or out-of-range data for any variable in the analysis.
Syntax		T-TEST GROUPS=Group('A' 'B')<br>/MISSING=ANALYSIS<br>/VARIABLES=height weight_pregavid bmi_pregavid weight_18years bmi_18years highest_weight highest_bmi<br>/ES DISPLAY(TRUE)<br>/CRITERIA=CI(.95).
Resources	Processor Time	00:00:00.00
	Elapsed Time	00:00:00.00

Group Statistics

	Group	N	Mean	Std. Deviation	Std. Error Mean
height	A	24	65.333	2.2393	.4571
	B	20	64.925	2.1538	.4816
weight_pregavid	A	24	133.71	12.274	2.505
	B	20	204.05	32.874	7.351
bmi_pregavid	A	24	22.027660419	1.8257240198	.37267435497
	B	20	34.084688932	5.6581961743	1.2652111276
weight_18years	A	24	124.08	16.251	3.317
	B	20	163.05	24.842	5.555
bmi_18years	A	24	20.455224330	2.6193300973	.53466851719
	B	20	27.265713820	4.5397107072	1.0151101740
highest_weight	A	24	142.00	15.294	3.122
	B	20	215.10	31.305	7.000
highest_bmi	A	24	23.394455669	2.2555665148	.46041558685
	B	20	35.983903589	5.7806234310	1.2925866944

Independent Samples Test

		Levene's Test for Equality of Variances		t-test for Equality of Means	
		F	Sig.	t	df
height	Equal variances assumed	.094	.761	.613	42
	Equal variances not assumed			.615	41.099
weight_pregavid	Equal variances assumed	22.683	<.001	-9.720	42
	Equal variances not assumed			-9.058	23.410
bmi_pregavid	Equal variances assumed	20.562	<.001	-9.861	42
	Equal variances not assumed			-9.141	22.301
weight_18years	Equal variances assumed	3.895	.055	-6.252	42
	Equal variances not assumed			-6.023	31.643
bmi_18years	Equal variances assumed	7.486	.009	-6.220	42
	Equal variances not assumed			-5.936	29.151
highest_weight	Equal variances assumed	9.328	.004	-10.100	42
	Equal variances not assumed			-9.537	26.446

Independent Samples Test

		t-test for Equality of Means		
		Significance		
		One-Sided p	Two-Sided p	Mean Difference
height	Equal variances assumed	.272	.543	.4083
	Equal variances not assumed	.271	.542	.4083
weight_pregavid	Equal variances assumed	<.001	<.001	-70.342
	Equal variances not assumed	<.001	<.001	-70.342
bmi_pregavid	Equal variances assumed	<.001	<.001	-12.05702851
	Equal variances not assumed	<.001	<.001	-12.05702851
weight_18years	Equal variances assumed	<.001	<.001	-38.967
	Equal variances not assumed	<.001	<.001	-38.967
bmi_18years	Equal variances assumed	<.001	<.001	-6.810489490
	Equal variances not assumed	<.001	<.001	-6.810489490
highest_weight	Equal variances assumed	<.001	<.001	-73.100
	Equal variances not assumed	<.001	<.001	-73.100

Independent Samples Test

		t-test for Equality of Means		
			95% Confidence Interval of the Difference	
		Std. Error Difference	Lower	Upper
height	Equal variances assumed	.6664	-.9365	1.7532
	Equal variances not assumed	.6640	-.9325	1.7492
weight_pregavid	Equal variances assumed	7.237	-84.947	-55.737
	Equal variances not assumed	7.766	-86.391	-54.292
bmi_pregavid	Equal variances assumed	1.2226767976	-14.52449019	-9.589566840
	Equal variances not assumed	1.3189561677	-14.79023492	-9.323822108
weight_18years	Equal variances assumed	6.233	-51.545	-26.389
	Equal variances not assumed	6.470	-52.151	-25.782
bmi_18years	Equal variances assumed	1.0949995136	-9.020287973	-4.600691007
	Equal variances not assumed	1.1473094999	-9.156473001	-4.464505980
highest_weight	Equal variances assumed	7.237	-87.706	-58.494
	Equal variances not assumed	7.665	-88.842	-57.358

Independent Samples Test

		Levene's Test for Equality of Variances		t-test for Equality of Means	
		F	Sig.	t	df
highest_bmi	Equal variances assumed	15.318	<.001	-9.827	42
	Equal variances not assumed			-9.175	23.811

Independent Samples Test

		t-test for Equality of Means		
		Significance		
		One-Sided p	Two-Sided p	Mean Difference
highest_bmi	Equal variances assumed	<.001	<.001	-12.58944792
	Equal variances not assumed	<.001	<.001	-12.58944792

Independent Samples Test

		t-test for Equality of Means		
		Std. Error Difference	95% Confidence Interval of the Difference	
			Lower	Upper
highest_bmi	Equal variances assumed	1.2810446299	-15.17470065	-10.00419519
	Equal variances not assumed	1.3721380671	-15.42259475	-9.756301088

Independent Samples Effect Sizes

		Standardizer[a]	Point Estimate	95% Confidence Interval	
				Lower	Upper
height	Cohen's d	2.2010	.186	-.410	.779
	Hedges' correction	2.2413	.182	-.403	.765
	Glass's delta	2.1538	.190	-.409	.784
weight_pregavid	Cohen's d	23.904	-2.943	-3.798	-2.071
	Hedges' correction	24.341	-2.890	-3.729	-2.033
	Glass's delta	32.874	-2.140	-3.026	-1.228
bmi_pregavid	Cohen's d	4.0383685517	-2.986	-3.847	-2.107
	Hedges' correction	4.1123165995	-2.932	-3.778	-2.069
	Glass's delta	5.6581961743	-2.131	-3.015	-1.222
weight_18years	Cohen's d	20.586	-1.893	-2.603	-1.167
	Hedges' correction	20.963	-1.859	-2.556	-1.146
	Glass's delta	24.842	-1.569	-2.329	-.783
bmi_18years	Cohen's d	3.6166643618	-1.883	-2.592	-1.159
	Hedges' correction	3.6828904294	-1.849	-2.545	-1.138
	Glass's delta	4.5397107072	-1.500	-2.247	-.729
highest_weight	Cohen's d	23.904	-3.058	-3.931	-2.168
	Hedges' correction	24.342	-3.003	-3.860	-2.129
	Glass's delta	31.305	-2.335	-3.269	-1.377
highest_bmi	Cohen's d	4.2311511569	-2.975	-3.835	-2.098
	Hedges' correction	4.3086293177	-2.922	-3.766	-2.061
	Glass's delta	5.7806234310	-2.178	-3.073	-1.257

a. The denominator used in estimating the effect sizes.
Cohen's d uses the pooled standard deviation.
Hedges' correction uses the pooled standard deviation, plus a correction factor.
Glass's delta uses the sample standard deviation of the control (i.e., the second) group.

Crosstabs

Notes

Output Created		02-APR-2026 16:24:18
Comments		
Input	Data	C:\Users\wahlquist\OneDrive - East Tennessee State University\Andy Clark\SugarMama\Data\Demographics_02APR2026.sav
	Active Dataset	DataSet1
	Filter	<none>
	Weight	<none>
	Split File	<none>
	N of Rows in Working Data File	44
Missing Value Handling	Definition of Missing	User-defined missing values are treated as missing.
	Cases Used	Statistics for each table are based on all the cases with valid data in the specified range(s) for all variables in each table.
Syntax		CROSSTABS<br>/TABLES=fishoilsupplement fish fish2 nut nuts2 flaxseed flaxseed2 other_supplement BY Group<br>/FORMAT=AVALUE TABLES<br>/CELLS=COUNT COLUMN<br>/COUNT ROUND CELL.
Resources	Processor Time	00:00:00.00
	Elapsed Time	00:00:00.01
	Dimensions Requested	2
	Cells Available	524245

Case Processing Summary

	Cases					
	Valid		Missing		Total	
	N	Percent	N	Percent	N	Percent
fishoilsupplement * Group	44	100.0%	0	0.0%	44	100.0%
fish * Group	44	100.0%	0	0.0%	44	100.0%
fish2 * Group	44	100.0%	0	0.0%	44	100.0%
nut * Group	44	100.0%	0	0.0%	44	100.0%
nuts2 * Group	44	100.0%	0	0.0%	44	100.0%
flaxseed * Group	44	100.0%	0	0.0%	44	100.0%
flaxseed2 * Group	44	100.0%	0	0.0%	44	100.0%
other_supplement * Group	43	97.7%	1	2.3%	44	100.0%

fishoilsupplement * Group Crosstabulation

			Group		Total
			A	B	
fishoilsupplement	Yes	Count	6	3	9
		% within Group	25.0%	15.0%	20.5%
	No	Count	18	17	35
		% within Group	75.0%	85.0%	79.5%
Total		Count	24	20	44
		% within Group	100.0%	100.0%	100.0%

fish * Group Crosstabulation

			Group		
			A	B	Total
fish	.0	Count	13	14	27
		% within Group	54.2%	70.0%	61.4%
	1.5	Count	1	1	2
		% within Group	4.2%	5.0%	4.5%
	2.0	Count	1	0	1
		% within Group	4.2%	0.0%	2.3%
	2.5	Count	1	2	3
		% within Group	4.2%	10.0%	6.8%
	3.0	Count	1	0	1
		% within Group	4.2%	0.0%	2.3%
	4.0	Count	3	1	4
		% within Group	12.5%	5.0%	9.1%
	6.0	Count	1	0	1
		% within Group	4.2%	0.0%	2.3%
	8.0	Count	1	1	2
		% within Group	4.2%	5.0%	4.5%
	8.5	Count	1	0	1
		% within Group	4.2%	0.0%	2.3%
	9.0	Count	0	1	1
		% within Group	0.0%	5.0%	2.3%
	10.0	Count	1	0	1
		% within Group	4.2%	0.0%	2.3%
Total		Count	24	20	44
		% within Group	100.0%	100.0%	100.0%

fish2 * Group Crosstabulation

			Group		
			A	B	Total
fish2	No	Count	13	14	27
		% within Group	54.2%	70.0%	61.4%
	Yes	Count	11	6	17
		% within Group	45.8%	30.0%	38.6%
Total		Count	24	20	44
		% within Group	100.0%	100.0%	100.0%

nut * Group Crosstabulation

			Group		
			A	B	Total
nut	.0	Count	3	1	4
		% within Group	12.5%	5.0%	9.1%
	1.5	Count	0	3	3
		% within Group	0.0%	15.0%	6.8%
	2.0	Count	1	2	3
		% within Group	4.2%	10.0%	6.8%
	2.5	Count	0	2	2
		% within Group	0.0%	10.0%	4.5%
	3.0	Count	2	0	2
		% within Group	8.3%	0.0%	4.5%
	3.5	Count	2	0	2
		% within Group	8.3%	0.0%	4.5%
	4.0	Count	2	0	2
		% within Group	8.3%	0.0%	4.5%
	4.5	Count	0	1	1
		% within Group	0.0%	5.0%	2.3%
	5.0	Count	4	0	4
		% within Group	16.7%	0.0%	9.1%
	5.5	Count	0	1	1
		% within Group	0.0%	5.0%	2.3%
	6.0	Count	1	0	1
		% within Group	4.2%	0.0%	2.3%
	10.0	Count	0	3	3
		% within Group	0.0%	15.0%	6.8%
	12.0	Count	1	0	1
		% within Group	4.2%	0.0%	2.3%
	13.5	Count	0	1	1
		% within Group	0.0%	5.0%	2.3%
	14.0	Count	1	0	1
		% within Group	4.2%	0.0%	2.3%
	15.0	Count	1	1	2
		% within Group	4.2%	5.0%	4.5%
	16.0	Count	1	0	1
		% within Group	4.2%	0.0%	2.3%
	20.0	Count	2	1	3
		% within Group	8.3%	5.0%	6.8%
	23.0	Count	1	0	1
		% within Group	4.2%	0.0%	2.3%

nut * Group Crosstabulation

			Group		Total
			A	B	
	30.0	Count	1	1	2
		% within Group	4.2%	5.0%	4.5%
	31.0	Count	1	3	4
		% within Group	4.2%	15.0%	9.1%
Total		Count	24	20	44
		% within Group	100.0%	100.0%	100.0%

nuts2 * Group Crosstabulation

			Group		Total
			A	B	
nuts2	No	Count	3	1	4
		% within Group	12.5%	5.0%	9.1%
	Yes	Count	21	19	40
		% within Group	87.5%	95.0%	90.9%
Total		Count	24	20	44
		% within Group	100.0%	100.0%	100.0%

flaxseed * Group Crosstabulation

			Group		Total
			A	B	
flaxseed	.0	Count	18	13	31
		% within Group	75.0%	65.0%	70.5%
	1.0	Count	1	0	1
		% within Group	4.2%	0.0%	2.3%
	2.0	Count	0	1	1
		% within Group	0.0%	5.0%	2.3%
	3.0	Count	0	1	1
		% within Group	0.0%	5.0%	2.3%
	4.0	Count	2	1	3
		% within Group	8.3%	5.0%	6.8%
	4.5	Count	1	0	1
		% within Group	4.2%	0.0%	2.3%
	9.0	Count	0	1	1
		% within Group	0.0%	5.0%	2.3%
	15.0	Count	1	0	1
		% within Group	4.2%	0.0%	2.3%
	20.0	Count	1	0	1
		% within Group	4.2%	0.0%	2.3%

flaxseed * Group Crosstabulation

			Group		Total
			A	B	
	30.0	Count	0	1	1
		% within Group	0.0%	5.0%	2.3%
	31.0	Count	0	2	2
		% within Group	0.0%	10.0%	4.5%
Total		Count	24	20	44
		% within Group	100.0%	100.0%	100.0%

flaxseed2 * Group Crosstabulation

			Group		Total
			A	B	
flaxseed2	No	Count	18	13	31
		% within Group	75.0%	65.0%	70.5%
	Yes	Count	6	7	13
		% within Group	25.0%	35.0%	29.5%
Total		Count	24	20	44
		% within Group	100.0%	100.0%	100.0%

other_supplement * Group Crosstabulation

			Group		Total
			A	B	
other_supplement	Yes	Count	7	4	11
		% within Group	29.2%	21.1%	25.6%
	No	Count	17	15	32
		% within Group	70.8%	78.9%	74.4%
Total		Count	24	19	43
		% within Group	100.0%	100.0%	100.0%

T-Test

Notes

Output Created		02-APR-2026 16:25:24
Comments		
Input	Data	C:\Users\wahlquist\OneDrive - East Tennessee State University\Andy Clark\SugarMama\Data\Demographics_02APR2026.sav
	Active Dataset	DataSet1
	Filter	<none>
	Weight	<none>
	Split File	<none>
	N of Rows in Working Data File	44
Missing Value Handling	Definition of Missing	User defined missing values are treated as missing.
	Cases Used	Statistics for each analysis are based on the cases with no missing or out-of-range data for any variable in the analysis.
Syntax		T-TEST GROUPS=Group ('A' 'B')<br>/MISSING=ANALYSIS<br>/VARIABLES=FVServings VitC Magnesium Potassium DietaryFiber<br>/ES DISPLAY(TRUE)<br>/CRITERIA=CI(.95).
Resources	Processor Time	00:00:00.02
	Elapsed Time	00:00:00.00

Group Statistics

	Group	N	Mean	Std. Deviation	Std. Error Mean
FVServings	A	24	3.9838	1.61947	.33057
	B	20	4.7885	2.36908	.52974
VitC	A	24	137.5537	40.23438	8.21281
	B	20	152.6500	59.19064	13.23543
Magnesium	A	24	342.867	72.3828	14.7751
	B	20	369.800	104.9127	23.4592
Potassium	A	24	3290.9833333	710.55240462	145.04090224
	B	20	3556.2200000	1035.1972808	231.47714899
DietaryFiber	A	24	16.9512	4.90148	1.00051
	B	20	18.7595	7.01582	1.56879

Independent Samples Test

		Levene's Test for Equality of Variances		t-test for Equality of Means	
		F	Sig.	t	df
FVServings	Equal variances assumed	4.820	.034	-1.333	42
	Equal variances not assumed			-1.289	32.595
VitC	Equal variances assumed	5.948	.019	-1.003	42
	Equal variances not assumed			-.969	32.472
Magnesium	Equal variances assumed	5.823	.020	-1.004	42
	Equal variances not assumed			-.971	32.800
Potassium	Equal variances assumed	5.873	.020	-1.004	42
	Equal variances not assumed			-.971	32.686
DietaryFiber	Equal variances assumed	5.672	.022	-1.003	42
	Equal variances not assumed			-.972	33.079

Independent Samples Test

		t-test for Equality of Means			
		Significance			
		One-Sided p	Two-Sided p	Mean Difference	Std. Error Difference
FVServings	Equal variances assumed	.095	.190	-.80475	.60365
	Equal variances not assumed	.103	.207	-.80475	.62442
VitC	Equal variances assumed	.161	.322	-15.09625	15.05147
	Equal variances not assumed	.170	.340	-15.09625	15.57648
Magnesium	Equal variances assumed	.161	.321	-26.9333	26.8222
	Equal variances not assumed	.169	.338	-26.9333	27.7243
Potassium	Equal variances assumed	.161	.321	-265.2366667	264.16505813
	Equal variances not assumed	.169	.339	-265.2366667	273.16393215
DietaryFiber	Equal variances assumed	.161	.321	-1.80825	1.80198
	Equal variances not assumed	.169	.338	-1.80825	1.86067

Independent Samples Test

		t-test for Equality of Means	
		95% Confidence Interval of the Difference	
		Lower	Upper
FVServings	Equal variances assumed	-2.02297	.41347
	Equal variances not assumed	-2.07575	.46625
VitC	Equal variances assumed	-45.47135	15.27885
	Equal variances not assumed	-46.80645	16.61395
Magnesium	Equal variances assumed	-81.0628	27.1961
	Equal variances not assumed	-83.3519	29.4853
Potassium	Equal variances assumed	-798.3433370	267.87000367
	Equal variances not assumed	-821.1958098	290.72247651
DietaryFiber	Equal variances assumed	-5.44479	1.82829
	Equal variances not assumed	-5.59348	1.97698

Independent Samples Effect Sizes

		Standardizer[a]	Point Estimate	95% Confidence Interval Lower	95% Confidence Interval Upper
FVServings	Cohen's d	1.99380	-.404	-1.001	.198
	Hedges' correction	2.03031	-.396	-.983	.195
	Glass's delta	2.36908	-.340	-.938	.268
VitC	Cohen's d	49.71337	-.304	-.899	.295
	Hedges' correction	50.62369	-.298	-.883	.290
	Glass's delta	59.19064	-.255	-.851	.347
Magnesium	Cohen's d	88.5909	-.304	-.899	.295
	Hedges' correction	90.2131	-.299	-.883	.289
	Glass's delta	104.9127	-.257	-.852	.345
Potassium	Cohen's d	872.50847104	-.304	-.899	.295
	Hedges' correction	888.48529367	-.299	-.883	.289
	Glass's delta	1035.1972808	-.256	-.852	.346
DietaryFiber	Cohen's d	5.95175	-.304	-.899	.295
	Hedges' correction	6.06073	-.298	-.883	.290
	Glass's delta	7.01582	-.258	-.853	.345

a. The denominator used in estimating the effect sizes.
Cohen's d uses the pooled standard deviation.
Hedges' correction uses the pooled standard deviation, plus a correction factor.
Glass's delta uses the sample standard deviation of the control (i.e., the second) group.

T-Test

Notes

Output Created		02-APR-2026 16:27:22
Comments		
Input	Data	C:\Users\wahlquist\OneDrive - East Tennessee State University\Andy Clark\SugarMama\Data\Demographics_02APR2026.sav
	Active Dataset	DataSet1
	Filter	<none>
	Weight	<none>
	Split File	<none>
	N of Rows in Working Data File	44
Missing Value Handling	Definition of Missing	User defined missing values are treated as missing.
	Cases Used	Statistics for each analysis are based on the cases with no missing or out-of-range data for any variable in the analysis.
Syntax		T-TEST GROUPS=Group ('A' 'B')<br>/MISSING=ANALYSIS<br>/VARIABLES=DietaryCholesterol Fat SaturatedFat PercentFat PercentSatFat<br>/ES DISPLAY(TRUE)<br>/CRITERIA=CI(.95).
Resources	Processor Time	00:00:00.00
	Elapsed Time	00:00:00.01

Group Statistics

	Group	N	Mean	Std. Deviation	Std. Error Mean
DietaryCholesterol	A	24	269.1250	49.88144	10.18201
	B	20	258.0100	50.42928	11.27633
Fat	A	24	106.600	15.3481	3.1329
	B	20	103.180	15.5167	3.4696
SaturatedFat	A	24	28.8900	5.62765	1.14874
	B	20	27.6360	5.68946	1.27220
PercentFat	A	24	37.775	3.8370	.7832
	B	20	36.920	3.8792	.8674
PercentSatFat	A	24	10.2133	1.57526	.32155
	B	20	9.8625	1.61064	.36015

Independent Samples Test

		Levene's Test for Equality of Variances		t-test for Equality of Means	
		F	Sig.	t	df
DietaryCholesterol	Equal variances assumed	.170	.682	.732	42
	Equal variances not assumed			.732	40.418
Fat	Equal variances assumed	.170	.682	.732	42
	Equal variances not assumed			.732	40.418
SaturatedFat	Equal variances assumed	.170	.682	.732	42
	Equal variances not assumed			.732	40.418
PercentFat	Equal variances assumed	.170	.682	.732	42
	Equal variances not assumed			.732	40.418
PercentSatFat	Equal variances assumed	.149	.701	.728	42
	Equal variances not assumed			.727	40.241

Independent Samples Test

		t-test for Equality of Means		
		Significance		
		One-Sided p	Two-Sided p	Mean Difference
DietaryCholesterol	Equal variances assumed	.234	.468	11.11500
	Equal variances not assumed	.234	.469	11.11500
Fat	Equal variances assumed	.234	.468	3.4200
	Equal variances not assumed	.234	.469	3.4200
SaturatedFat	Equal variances assumed	.234	.468	1.25400
	Equal variances not assumed	.234	.469	1.25400
PercentFat	Equal variances assumed	.234	.468	.8550
	Equal variances not assumed	.234	.469	.8550
PercentSatFat	Equal variances assumed	.235	.471	.35083
	Equal variances not assumed	.236	.472	.35083

Independent Samples Test

		t-test for Equality of Means		
		Std. Error Difference	95% Confidence Interval of the Difference	
			Lower	Upper
DietaryCholesterol	Equal variances assumed	15.17761	-19.51467	41.74467
	Equal variances not assumed	15.19305	-19.58142	41.81142
Fat	Equal variances assumed	4.6700	-6.0045	12.8445
	Equal variances not assumed	4.6748	-6.0251	12.8651
SaturatedFat	Equal variances assumed	1.71235	-2.20165	4.70965
	Equal variances not assumed	1.71409	-2.20919	4.71719
PercentFat	Equal variances assumed	1.1675	-1.5011	3.2111
	Equal variances not assumed	1.1687	-1.5063	3.2163
PercentSatFat	Equal variances assumed	.48181	-.62149	1.32316
	Equal variances not assumed	.48280	-.62477	1.32644

Independent Samples Effect Sizes

		Standardizer[a]	Point Estimate	95% Confidence Interval	
				Lower	Upper
DietaryCholesterol	Cohen's d	50.13001	.222	-.375	.816
	Hedges' correction	51.04796	.218	-.368	.801
	Glass's delta	50.42928	.220	-.380	.815
Fat	Cohen's d	15.4246	.222	-.375	.816
	Hedges' correction	15.7071	.218	-.368	.801
	Glass's delta	15.5167	.220	-.380	.815
SaturatedFat	Cohen's d	5.65569	.222	-.375	.816
	Hedges' correction	5.75926	.218	-.368	.801
	Glass's delta	5.68946	.220	-.380	.815
PercentFat	Cohen's d	3.8562	.222	-.375	.816
	Hedges' correction	3.9268	.218	-.368	.801
	Glass's delta	3.8792	.220	-.380	.815
PercentSatFat	Cohen's d	1.59136	.220	-.376	.814
	Hedges' correction	1.62050	.216	-.369	.800
	Glass's delta	1.61064	.218	-.382	.812

a. The denominator used in estimating the effect sizes.
Cohen's d uses the pooled standard deviation.
Hedges' correction uses the pooled standard deviation, plus a correction factor.
Glass's delta uses the sample standard deviation of the control (i.e., the second) group.
