# Supplementary material for: Maternal Pre-Pregnancy Body Mass Index and Its Impact on Short- and Long-Chain Fatty Acid and Microbiome Profiles of Human Breast Milk in Caucasian Women of Northeast Tennessee
Source: Nutrients. 2026 Jun 12;18(12):1917. doi: 10.3390/nu18121917 (PMC13304685; doi:10.3390/nu18121917)
Supplement: Supplementary file 1 [file nutrients-18-01917-s001.zip › Proximate Analysis_15APR2026.pdf]

T-Test

Notes

Output Created		15-APR-2026 11:00:13
Comments		
Input	Data	C:\Users\wahlquist\OneDrive - East Tennessee State University\Andy Clark\SugarMama\Data\Proximate_15APR2026.sav
	Active Dataset	DataSet1
	Filter	<none>
	Weight	<none>
	Split File	<none>
	N of Rows in Working Data File	44
Missing Value Handling	Definition of Missing	User defined missing values are treated as missing.
	Cases Used	Statistics for each analysis are based on the cases with no missing or out-of-range data for any variable in the analysis.
Syntax		T-TEST GROUPS=Group ('A' 'B') /MISSING=ANALYSIS /VARIABLES=DryWeight Caloires Protein Fat Ashing /ES DISPLAY(TRUE) /CRITERIA=CI(.95).
Resources	Processor Time	00:00:00.00
	Elapsed Time	00:00:00.01

```
[DataSet1] C:\Users\wahlquist\OneDrive - East Tennessee State University\Andy Clark\SugarMama\Data\Proximat
e_15APR2026.sav
```

Group Statistics

	Group	N	Mean	Std. Deviation	Std. Error Mean
% Dry Weight	A	21	12.3352	1.22176	.26661
	B	15	12.4820	1.07882	.27855
Calories/g	A	24	5581.7558000	301.30295057	61.50320724
	B	20	5562.9262050	403.02810075	90.11982301
% Protein	A	24	9.79979	1.532112	.312741
	B	20	8.92925	2.197072	.491280
% Fat	A	24	39.604	7.5800	1.5473
	B	20	43.850	9.7577	2.1819
% inorganic material	A	24	1.4479	.44220	.09026
	B	20	1.4750	.34317	.07673

Independent Samples Test

		Levene's Test for Equality of Variances		t-test for Equality of .
		F	Sig.	t
% Dry Weight	Equal variances assumed	.059	.809	-.373
	Equal variances not assumed			-.381
Calories/g	Equal variances assumed	1.818	.185	.177
	Equal variances not assumed			.173
% Protein	Equal variances assumed	2.168	.148	1.544
	Equal variances not assumed			1.495
% Fat	Equal variances assumed	2.980	.092	-1.624
	Equal variances not assumed			-1.587
% inorganic material	Equal variances assumed	1.986	.166	-.223
	Equal variances not assumed			-.229

Independent Samples Test

		t-test for Equality of Means		
			Significance	
		df	One-Sided p	Two-Sided p
% Dry Weight	Equal variances assumed	34	.356	.712
	Equal variances not assumed	32.379	.353	.706
Calories/g	Equal variances assumed	42	.430	.860
	Equal variances not assumed	34.617	.432	.864
% Protein	Equal variances assumed	42	.065	.130
	Equal variances not assumed	33.037	.072	.144
% Fat	Equal variances assumed	42	.056	.112
	Equal variances not assumed	35.498	.061	.121
% inorganic material	Equal variances assumed	42	.412	.824
	Equal variances not assumed	41.818	.410	.820

Independent Samples Test

		t-test for Equality of Means		
				95% Confidence Interval of the ...
		Mean Difference	Std. Error Difference	Lower
% Dry Weight	Equal variances assumed	-.14676	.39385	-.94716
	Equal variances not assumed	-.14676	.38558	-.93180
Calories/g	Equal variances assumed	18.82959500	106.26825918	-195.62843443
	Equal variances not assumed	18.82959500	109.10649385	-202.75603355
% Protein	Equal variances assumed	.870542	.563921	-.267497
	Equal variances not assumed	.870542	.582377	-.314263
% Fat	Equal variances assumed	-4.2458	2.6139	-9.5209
	Equal variances not assumed	-4.2458	2.6748	-9.6733
% inorganic material	Equal variances assumed	-.02708	.12124	-.27176
	Equal variances not assumed	-.02708	.11847	-.26620

Independent Samples Test

		t-test for Equality of Means<br>95% Confidence Interval of the ...<br>Upper
% Dry Weight	Equal variances assumed	.65364
	Equal variances not assumed	.63827
Calories/g	Equal variances assumed	233.28762443
	Equal variances not assumed	240.41522355
% Protein	Equal variances assumed	2.008580
	Equal variances not assumed	2.055346
% Fat	Equal variances assumed	1.0293
	Equal variances not assumed	1.1816
% inorganic material	Equal variances assumed	.21759
	Equal variances not assumed	.21203

Independent Samples Effect Sizes

		Standardizer[a]	Point Estimate	95% Confidence Interval<br>Lower	 Upper
% Dry Weight	Cohen's d	1.16503	-.126	-.788	.538
	Hedges' correction	1.19154	-.123	-.771	.526
	Glass's delta	1.07882	-.136	-.798	.531
Calories/g	Cohen's d	350.99250821	.054	-.540	.647
	Hedges' correction	357.41966076	.053	-.530	.635
	Glass's delta	403.02810075	.047	-.547	.640
% Protein	Cohen's d	1.862569	.467	-.137	1.066
	Hedges' correction	1.896675	.459	-.135	1.047
	Glass's delta	2.197072	.396	-.215	.998
% Fat	Cohen's d	8.6335	-.492	-1.092	.114
	Hedges' correction	8.7916	-.483	-1.072	.112
	Glass's delta	9.7577	-.435	-1.039	.179
% inorganic material	Cohen's d	.40044	-.068	-.661	.526
	Hedges' correction	.40778	-.066	-.649	.517
	Glass's delta	.34317	-.079	-.672	.516

a. The denominator used in estimating the effect sizes.
Cohen's d uses the pooled standard deviation.
Hedges' correction uses the pooled standard deviation, plus a correction factor.
Glass's delta uses the sample standard deviation of the control (i.e., the second) group.
