# Supplementary material for: Maternal Pre-Pregnancy Body Mass Index and Its Impact on Short- and Long-Chain Fatty Acid and Microbiome Profiles of Human Breast Milk in Caucasian Women of Northeast Tennessee
Source: Nutrients. 2026 Jun 12;18(12):1917. doi: 10.3390/nu18121917 (PMC13304685; doi:10.3390/nu18121917)
Supplement: Supplementary file 1 [file nutrients-18-01917-s001.zip › Supplemental Figure S1.pdf]

Supplemental Figure S1- Demographic and Health Survey

Lactation Study
Demographic and Health survey

Date: _______________ Participant PIN: _______________

Demographic Information

1. Date of birth:

   _____/ 1 9 __ __
   Month/4-digit year

2. Race/Hispanic Origin:

   - ☐ 1 Mexican American
   - ☐ 2 Other Hispanic
   - ☐ 3 Non-Hispanic White
   - ☐ 4 Non-Hispanic Black
   - ☐ 5 Other Race – Including Multi-Racial

3. Marital status:

   - ☐ 1 Married
   - ☐ 2 Widowed
   - ☐ 3 Divorced
   - ☐ 4 Separated
   - ☐ 5 Never married
   - ☐ 6 Living with partner
   - ☐ 77 Refused
   - ☐ 99 Don't know

4. What is the highest grade or level of school you have completed or highest degree received?

   - ☐ 1 Less than 9th grade
   - ☐ 2 9-11th grade (includes 12th grade with no diploma)
   - ☐ 3 High school graduate/GED or equivalent
   - ☐ 4 Some college or AA degree
   - ☐ 5 College graduate or above
   - ☐ 77 Refused
   - ☐ 99 Don't know

5. Employment Status:

   - ☐ 1 Work for someone else full time
   - ☐ 2 Temporarily unemployed
   - ☐ 3 Self-employed
   - ☐ 4 Work for someone else part-time
   - ☐ 5 Retired, not employed
   - ☐ 6 Student, disabled, etc., not employed
   - ☐ 7 Full time homemaker
   - ☐ 77 Refused
   - ☐ 99 Don't know

Lactation Study
Demographic and Health survey

Date: _______________ Participant PIN: _______________

6. What was your total household income for the last completed year, before taxes and deductions?

- ☐ 1 $0 to $4,999
- ☐ 2 $5,000 to $9,999
- ☐ 3 $10,000 to 14,999
- ☐ 4 $15,000 to 19,999
- ☐ 5 $20,000 to $24,999
- ☐ 6 $25,000 to $34,999
- ☐ 7 $35,000 to $44,999
- ☐ 8 $45,000 to $54,999
- ☐ 9 $55,000 to $64,999
- ☐ 10 $65,000 to $74,999
- ☐ 11 $75,000 to 99,999
- ☐ 12 $100,000 or more
- ☐ 77 Refused
- ☐ 99 Don't know

Health Care Services Utilization

7. Who provided your prenatal care?

- ☐ 1 An obstetrician
- ☐ 2 A family doctor, general practitioner, internist, or other physician
- ☐ 3 A midwife or nurse midwife
- ☐ 4 Another type of health care provider
- ☐ 5 I was not getting prenatal care from a health professional

8. Which type of health professional was your birth attendant (someone who delivered your baby)?

- ☐ 1 An obstetrician
- ☐ 2 A family doctor, general practitioner, internist, or other physician
- ☐ 3 A midwife or nurse midwife
- ☐ 4 Another type of health care provider
- ☐ 5 No health professional was present

Tobacco Smoking

9. Which sentence best describes your smoking pattern during the year before your child was born?

- ☐ 1 I did not smoke
- ☐ 2 I smoked but stopped before pregnancy began
- ☐ 3 I smoked, and stopped during the first 3 months of pregnancy
- ☐ 4 I smoked through the first 3 months but stopped before my child was born
- ☐ 5 I smoked throughout the year

10. During the year before your child was born, how frequently did you come in contact with family, friends or co-workers who smoked?

- ☐ 1 Never

- ☐ 2 Sometimes
- ☐ 3 Often
- ☐ 4 Always

12. On the average, how many cigarettes do you smoke a day now? (Write in 0 if you do not smoke).
____________ CIGARETTES PER DAY

13. How many people not including yourself smoke inside your home most days? (Include family members, friends, and anyone else.)

- ☐ 1 0
- ☐ 2 1
- ☐ 3 1
- ☐ 4 3
- ☐ 5 4 or more

Physical Activity

14. Think about the time you spend doing work. Think of work as the things that you do such as paid or unpaid work, studying or training, household chores, and yard work. Does your work involve moderate-intensity activity that causes small increases in breathing or heart rate such as brisk walking or carrying light loads for at least 10 minutes continuously?

- ☐ 1 Yes
- ☐ 2 No (Go to question 17)

15. In a typical week, on how many days do you do moderate-intensity activities as part of your work?

Number of days ____

16. How much time do you spend doing moderate-intensity activities at work on a typical day?

Number of minutes or hours ____

17. In a typical week do you do any moderate-intensity sports, fitness or recreational activities that cause a small increase in breathing or heart rate such as bicycling, swimming, or golf for at least 10 minutes continuously?

- ☐ 1 Yes
- ☐ 2 No (Go to question 20)

18. In a typical week, on how many days do you do moderate-intensity sports, fitness or recreational activities?

Number of days ____

19. How much time do you spend doing moderate-intensity sports, fitness or recreational activities on a typical day?

Number of minutes or hours ____

Lactation Study
Demographic and Health survey

Date: _______________ Participant PIN: _______________

Pregnancy Questions

20. Including this most recent pregnancy, how many times have you been pregnant?

- ☐ 1 1
- ☐ 2 2
- ☐ 3 3
- ☐ 4 4 or more

21. Did you take prenatal vitamins?

- ☐ 1 Yes
- ☐ 2 No

22. Are you taking prenatal vitamins now?

- ☐ 1 Yes
- ☐ 2 No (Go to question 18)

23. Name of your prenatal vitamin ___________________________

24. Birth weight of baby (lbs/oz) _____________________________

25. Sex of baby

- ☐ 1 Male
- ☐ 2 Female

26. Lactation sample (number days after giving birth) ____________________

Weight Questions

27. What was your pre-gravid weight (weight before most recent pregnancy) ______________lbs

28. Weight at age 18 years:

- ☐ 1 _______________lbs
- ☐ 2 Not applicable

29. Highest weight ever obtained when not pregnant ___________________lbs

30. Current Height: ___________________ft/inches

Lactation Study
Demographic and Health survey

Date: ______________ Participant PIN: ______________

Dietary Information

31. Do you take a daily fish oil/krill oil supplement?

- ☐ 1 Yes
- ☐ 2 No

32. Do you routinely eat fish?

- ☐ 1 Yes, How frequently? ______________ times per month
- ☐ 2 No

33. Do you eat nuts?

- ☐ 1 Yes, How frequently? ______________ times per month
- ☐ 2 No (Go to Question 29)

34. What types of nuts do you consume? ______________

35. Do you eat flaxseed or take flaxseed oil?

- ☐ 1 Yes, How frequently? ______________ times per month
- ☐ 2 No

36. Do you take any dietary supplements?

- ☐ 1 Yes, Please list ______________
- ☐ 2 No
