# Supplementary material for: Maternal Pre-Pregnancy Body Mass Index and Its Impact on Short- and Long-Chain Fatty Acid and Microbiome Profiles of Human Breast Milk in Caucasian Women of Northeast Tennessee
Source: Nutrients. 2026 Jun 12;18(12):1917. doi: 10.3390/nu18121917 (PMC13304685; doi:10.3390/nu18121917)
Supplement: Supplementary file 1 [file nutrients-18-01917-s001.zip › Supplemental Figure S2.pdf]

## Supplemental Figure S2- BLOCK Dietary Fruit & Vegetable Screener

# Dietary Fruit-Vegetable-Fiber Screener©

**Name :** ____________________

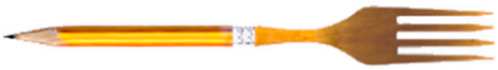

**Age:** ____

**Sex:** ○ Male ○ Female

**Think about your eating habits over the past month. About how often do you eat each of the following foods? Remember breakfast, lunch, dinner, snacks and eating out. Mark one bubble for each food.**

| **Fruits and Vegetables and Fiber** | (0) Less than 1 / WEEK | (1) Once a WEEK | (2) 2-3 times a WEEK | (3) 4-6 times a WEEK | (4) Once a DAY | (5) 2+ a DAY | **Score** |
|---|---|---|---|---|---|---|---|
| (1) Fruit juice, like orange, apple, grape, fresh, frozen or canned (Not sodas or other drinks.) | ○ | ○ | ○ | ○ | ○ | ○ | ______ |
| (2) How often do you eat any fruit, fresh or canned? (Not counting juice.) | ○ | ○ | ○ | ○ | ○ | ○ | ______ |
| (3) Vegetable juice, like tomato juice, V-8, carrot | ○ | ○ | ○ | ○ | ○ | ○ | ______ |
| (4) Green salad | ○ | ○ | ○ | ○ | ○ | ○ | ______ |
| (5) Potatoes, any kind, including baked, mashed or French fried | ○ | ○ | ○ | ○ | ○ | ○ | ______ |
| (6) Vegetable soup, or stew with vegetables | ○ | ○ | ○ | ○ | ○ | ○ | ______ |
| (7) Any other vegetables, including string beans, peas, corn, broccoli or any other kind | ○ | ○ | ○ | ○ | ○ | ○ | ______ |
| (8) Fiber cereals like Raisin Bran, Shredded Wheat or Fruit-n-Fiber | ○ | ○ | ○ | ○ | ○ | ○ | ______ |
| (9) Beans such as baked beans, pinto, kidney, or lentils (not green beans) | ○ | ○ | ○ | ○ | ○ | ○ | ______ |
| (10) Dark bread such as whole wheat or rye | ○ | ○ | ○ | ○ | ○ | ○ | ______ |
| | **Fruit-Vegetable Score (Sum for items 1-7) =** | | | | | | ______ |
| | **Fruit-Veg-Beans Score (Sum of items 1-10) =** | | | | | | ______ |

**BLOCK DIETARY DATA SYSTEMS**
*www.nutritionquest.com*
(510) 704-8514
