# Supplementary material for: Maternal Pre-Pregnancy Body Mass Index and Its Impact on Short- and Long-Chain Fatty Acid and Microbiome Profiles of Human Breast Milk in Caucasian Women of Northeast Tennessee
Source: Nutrients. 2026 Jun 12;18(12):1917. doi: 10.3390/nu18121917 (PMC13304685; doi:10.3390/nu18121917)
Supplement: Supplementary file 1 [file nutrients-18-01917-s001.zip › Supplemental Figure S3.pdf]

Supplemental Figure S3- BLOCK Dietary Fat Screener

Dietary Fat Screener©

Name : ______________________

Age: ____

Sex: O Male O Female

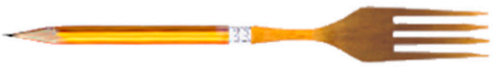

Think about your eating habits over the past month. About how often do you eat each of the following foods? Remember breakfast, lunch, dinner, snacks and eating out. Mark one bubble for each food.

Meals and Snacks	(0) 1/ MONTH or less	(1) 2-3 times a MONTH	(2) 1-2 times a WEEK	(3) 3-4 times a WEEK	(4) 5+ times a WEEK	Score
Hamburgers, ground beef, meat burritos, tacos	O	O	O	O	O	______
Beef or pork, such as steaks, roasts, ribs, or in sandwiches	O	O	O	O	O	______
Fried chicken	O	O	O	O	O	______
Hot dogs, or Polish or Italian sausage	O	O	O	O	O	______
Cold cuts, lunch meats, ham (not low-fat)	O	O	O	O	O	______
Bacon or breakfast sausage	O	O	O	O	O	______
Salad dressings (not low-fat)	O	O	O	O	O	______
Margarine, butter or mayo on bread or potatoes	O	O	O	O	O	______
Margarine, butter or oil in cooking	O	O	O	O	O	______
Eggs (not Egg Beaters or just egg whites)	O	O	O	O	O	______
Pizza	O	O	O	O	O	______
Cheese, cheese spread (not low-fat)	O	O	O	O	O	______
Whole milk	O	O	O	O	O	______
French fries, fried potatoes	O	O	O	O	O	______
Corn chips, potato chips, popcorn, crackers	O	O	O	O	O	______
Doughnuts, pastries, cake, cookies (not low-fat)	O	O	O	O	O	______
Ice cream (not sherbet or non-fat)	O	O	O	O	O	______

Fat Score = __________

NUTRITIONQUEST / BLOCK DIETARY DATA SYSTEMS
www.nutritionquest.com
510.704.8514
